# MultiFRAGing: Rapid and Simultaneous Genotyping of Multiple Alleles in a Single Reaction

**DOI:** 10.1038/s41598-020-59986-1

**Published:** 2020-02-21

**Authors:** Cassidy Petree, Gaurav K. Varshney

**Affiliations:** Genes & Human Disease Research Program, Oklahoma Medical Research Foundation, Oklahoma City, OK 73104 USA

**Keywords:** Mutagenesis, DNA sequencing

## Abstract

Powerful and simple, RNA-guided CRISPR/Cas9 technology is a versatile genome editing tool that has revolutionized targeted mutagenesis. CRISPR-based genome editing has enabled large-scale functional genetic studies through the generation of gene knockouts in a variety of model organisms including zebrafish, and can be used to target multiple genes simultaneously. One of the challenges associated with the large scale application of this technique to zebrafish is the lack of a cost-effective method by which to identify mutants. To address this, we optimized the high-throughput, high-resolution fluorescent PCR-based fragment analysis method to develop MultiFRAGing - a robust and cost-effective method to genotype multiple targets in a single reaction. Our approach can identify indels in up to four targets from a single reaction, which represents a four-fold increase in genotyping throughput. This method can be used by any laboratory with access to capillary electrophoresis-based sequencing equipment.

## Introduction

Following completion of the human genome sequencing project, identification of candidate disease genes has been the focus of much genetic research. With the development of less expensive sequencing technologies, such genes are being discovered at a rapid rate, but functional validation remains slow. Most of the knowledge of gene function has been generated using gene knockout technology in model organisms^1^.

The zebrafish (*Danio rerio*) has become a popular model organism for many reasons including high fecundity, optically transparent embryos and larvae, external development and the ease with which various types of genetic manipulation can be performed^2^. A large number of zebrafish mutants have been generated using a variety of random mutagenesis approaches involving either the chemical mutagen N-ethyl-N-nitrosourea (ENU) or insertional mutagens (Retroviruses, Transposons)^2^. Recent progress in the transformative, targeted, and simple RNA-guided CRISPR/Cas9-based genome editing method has expedited genetic manipulation in many systems, including zebrafish^3–5^. In addition to CRISPR/Cas9, Transcription Activator-like Effector Nuclease(TALENS), and Zinc Finger Nucleases (ZFNs) are other targeted mutagenesis methods being used to generate knockouts for the purposes of developing disease models and understanding disease pathology in zebrafish^2,6^. Recently, the structure-guided endonuclease (SGN) - a DNA-guided genome editing tool that uses flap endonuclease 1 (FEN-1) fused to the Fok1 endonuclease - was also used to generate large deletions in the zebrafish genome^7,8^. While CRISPR/Cas9 is a RNA-guided endonuclease and TALENs and ZFNSs are engineered proteins, the SGN nuclease functions in a similar manner: it induces a double stranded break (DSB) at a DNA target site which is then repaired by an error-prone, non-homologous end-joining method that often leaves insertions and/or deletions during the repair.

Because of its simplicity, low-cost and ability to target multiple sites simultaneously, CRISPR/Cas9 is becoming most popular genome editing tool, and many workflows are available for generating large numbers of gene knockouts^9–13^. While the generation of knockouts is relatively straight forward, the identification of mutations in a high-throughput, affordable manner remains a challenge. More than 70% of the indels generated by CRISPR/Cas9 (or TALENs/ZFNs) are less than 20 bp, making genotyping challenging^9^. The most sensitive method used to identify indels involves amplification of the target region followed by cloning and sequencing - a labor intensive and time-consuming process not amenable to high-throughput technology. A number of other methods including high-resolution melt analysis (HRMA), melting curve analysis, restriction fragment length polymorphism (RFLP) analysis, PAGE-based screening, and the Surveyor assay are suitable to identify mutations on a small scale^14–18^. We and others previously adopted a high-throughput, high-resolution fragment analysis-based method to genotype CRISPR-induced alleles^9,19,20^, and later demonstrated that it can also be used to determine guide RNA activities *in vivo*^21^. Fragment analysis involves generation of double-stranded fluorescently labeled fragments using PCR, and subsequent separation by size using capillary electrophoresis; software determines the relative size of each fluorescently labeled fragment by comparison with a size standard to generate the genotype of each amplicon^22^.

Over the past 10 years, zebrafish has emerged as a preferred model organism to study various human diseases, and the use of CRISPR/Cas9 is fueling this growth^6^. As the throughput of CRISPR/Cas9- mediated mutagenesis increases, researchers are able to target multiple genes simultaneously, and the development of a multiplex genotyping method to reduce both cost and labor is needed. Here, we detail the development of MultiFRAGing, a multiplexing fragment analysis pipeline that could genotype up to four targets in a single reaction, increasing the throughput up to 4-fold while significantly reducing cost.

## Materials and Methods

### Ethics statement, and zebrafish care

The zebrafish experiments were carried out in compliance with the National Institutes of Health guidelines for animal handling and research under Oklahoma Medical Research Foundation (OMRF) Institutional Animal Care and Use Committee (IACUC) approved protocol 17-01. Zebrafish were housed in an AAALAC (Association for Assessment and Accreditation of Laboratory Animal Care) accredited facility. All zebrafish handling, embryo care, and microinjections were performed according to procedures described in The Zebrafish Book^23^. Wildtype (WT) zebrafish strain TAB-5 was used for all experiments. Zebrafish embryos were maintained in E3 embryo medium with 0.00002% methylene blue and raised at 28 °C.

### Generation of mutant lines using CRISPR/Cas9 in zebrafish

The guide RNAs (sgRNAs), Cas9 mRNA synthesis, and microinjections were carried out as described earlier^9,10^. Injected eggs were raised to the adulthood to generate founder fish. Six to eight founder fish were outcrossed with the wild type fish to generate heterozygous progenies (F_1_). Progenies from founders carrying mutations were raised to adulthood to generate the F_1_ generation, adults of which were genotyped using fragment analysis as previously described^9,10,22^.

To generate mutant lines with multiple alleles for multiplex genotyping, homozygous *dfnb31a*−/−*; dfnb31b*−/− double mutants were crossed with *grhl2a*−/−*;* *grhl2b*−/− double mutants. The progenies were raised to adulthood, and fish carrying five different alleles (*dfnb31a T1, dfnb31b T1, grhl2a T1, grhl2a T2, and grhl2b T1)* in four genes were used to establish this method.

### Primer design for fragment analysis

Primers were designed to amplify amplicons 180–300 bp in length, usually keeping the target site in the middle of the amplified fragments. In principle, fluorescently labelled amplicons can be generated by labelling one primer with a fluorophore, but this approach is not cost effective as designing fluorescent labeled primers for each target is expensive. To make it more affordable, we previously modified the method to include an adapter sequence (M13Fwd or T3 or SP6) to tail the 5′-end of gene-specific forward primers^9^. A third fluorescently labeled primer (M13Fwd-FAM, T3-TAMRA or SP6-HEX) was designed for use with gene-specific primers. This strategy avoids the cost associated with the fluorescent labeling of individual primers. The list of primers is listed in Table 1.

**Table 1:** Primer sequences used in this study.

Primer Name	Sequence
M13Fwd	TGTAAAACGACGGCCAGT
SP6	ATTTAGGTGACACTATAG
T3	ATTAACCCTCACTAAAGG
M13Fwd-FAM	/56-FAM/TGTAAAACGACGGCCAGT
SP6-HEX	/5HEX/ATTTAGGTGACACTATAG
T3-TAMRA	/56-TAMN/ATTAACCCTCACTAAAGG
M-*grhl2a* T2-F-FAM	tgtaaaacgacggccagtTCCGAACACCACCATCACTA
M-*grhl2a* T2-F-HEX	atttaggtgacactatagTCCGAACACCACCATCACTA
M-*grhl2a* T2-F-TAMRA	attaaccctcactaaaggTCCGAACACCACCATCACTA
M-*grhl2a* T2-R	gtgtcttATTGAAGCAAGCCGTTCTGT
M-*grhl2b* T1-F-FAM	tgtaaaacgacggccagtGAAACAGCAGCCAAATGGAG
M-*grhl2b* T1-F-HEX	atttaggtgacactatagGAAACAGCAGCCAAATGGAG
M-*grhl2b* T1-F-TAMRA	attaaccctcactaaaggGAAACAGCAGCCAAATGGAG
M-*grhl2b* T1-R	gtgtcttGTCCTGTAGTGTCCCCCTGA
M-*dfnb31a* T1-F-FAM	tgtaaaacgacggccagtGTGCTGATGCTGTCAGGAGA
M-*dfnb31a* T1-F-HEX	atttaggtgacactatagGTGCTGATGCTGTCAGGAGA
M-*dfnb31a* T1-F-TAMRA	attaaccctcactaaaggGTGCTGATGCTGTCAGGAGA
M-*dfnb31a* T1-R	gtgtcttGCTCGGATCAGCTTCTGTTT
M-*dfnb31b* T1-F-FAM	tgtaaaacgacggccagtCACCTTGACTGCCTCTCCAT
M-*dfnb31b* T1-F-HEX	atttaggtgacactatagCACCTTGACTGCCTCTCCAT
M-*dfnb31b* T1-F-TAMRA	attaaccctcactaaaggCACCTTGACTGCCTCTCCAT
M-*dfnb31b* T1-R	gtgtcttGGCTTCTGTTTTCAGCACCT

In order to avoid stutter peaks in genotyping, we added a 7-nucleotide tag (PIGTAIL) at the end of the gene-specific reverse primer. Taq DNA polymerases often catalyzes the non-templated addition of nucleotides to the 3′ end of PCR amplicons in a primer-specific activity that can introduce errors in accurate genotyping. Brownstein *et al*. demonstrated that the inclusion of a GTGTCTT sequence (PIGTAIL) in the reverse primer could suppress this activity^24^. The PIGTAIL was later adopted by Sood *et al*. in genotyping indels in zebrafish^22^. The sizes of fluorescent amplicons are calculated as follows: size of amplicon amplified by gene-specific primers + (size of tailed sequence attached to forward primer + 7 bp PIGTAIL sequence).

Multiplexing can be done in following ways:

1. Amplicons separated by two/three different sizes amplified in a single reaction.
2. Amplicons separated by two/three different colors amplified in a single reaction.
3. Amplicons separated by three/four different sizes amplified separately, and pooled together for capillary electrophoresis.
4. Amplicons separated by three different colors and two sizes amplified separately, and pooled together for capillary electrophoresis.

### Genomic DNA extraction

Genomic DNA was extracted using a previously described method^10^. Fins were clipped from heterozygous adults, tail pieces were dissolved in 30 µl 50 mM NaOH, heated to 95 °C for 20 minutes to completely dissolve all tissue, vortexed and centrifuged. 30 µl 100 mM Tris-HCl (pH 8.0) was added as a neutralization solution. DNA was diluted 10X with nuclease-free water. Extracted DNA can be stored at −20 °C for up to six months. For 2–5dpf embryos, we recommend using 10 µl of NaOH and 10 µl Tris-HCL and DNA should be diluted to 50X.

### Multiplex PCR setup

First, all three primers were mixed together as follows:

- 5 µL 100 µM Fluorescent Primer
- 3 µL 100 µM Gene-specific forward Primer
- 5 µL 100 µM Gene-specific reverse Primer
- 487 µL water

Any polymerase can be used for PCR, we tested multiple polymerases with variable results, reactions and conditions for two different polymerases are as follows:

- PCR reactions using **Platinum Taq** Polymerase were set-up in 20 µl final volume as follows:
  2 µL 10x Buffer
  0.6 µL MgCl2
  0.4 µl dNTP Mix (10 m M, New England Biolabs, Cat # N0447L)
  1.2 µL Primer mix (for each)
  0.16 µl Platinum Taq Polymerase (ThermoFisher Scientific, Cat # 100021275)
  2 µL DNA Template
  To 20 µL with Water
  PCR was performed using the following conditions:
  5 min denaturation at 94 °C followed by 34 cycles of: 94 °C for 30 sec, 57 °C for 30 sec, and 72 °C for 30 sec; and 5 min final extension at 72 °C.
- PCR reactions using Qiagen **HotStarTaq** Polymerase were set-up in 20 µl final volume as follows:
  2 µL 10x Buffer
  0.4 µl dNTP Mix (10 m M, New England Biolabs, Cat # N0447L)
  2 µL Primer mix (for each)
  0.1 µl Qiagen HotStar Taq Plus (Qiagen LLC, Cat # 203609)
  2 µL DNA Template
  To 20 µL with Water
  PCR was performed using the following conditions:
  15 min denaturation at 95 °C, 30 seconds denaturation at 94 °C followed by 34 cycles of: 94 °C for 30 sec, 55 °C for 30 sec, and 72 °C for 30 sec; and 5 min final extension at 72 °C.

Successful PCR amplification was confirmed by electrophoresis on 2% agarose gels.

### Fragment analysis by capillary electrophoresis

Here, fluorescent PCR fragments were separated by capillary electrophoresis on a Genetic Analyzer (ABI 3500-XL), although fragment analysis can be performed on any ABI Genetic Analyzer platform. A detailed protocol for fragment analysis has been described earlier^10^. Briefly, double-stranded, fluorescently-labelled PCR fragments were mixed with a size standard. We used GenScan 400 HD ROX size standard (ThermoFisher Scientific, Cat # 402985) as an internal size marker: ROX was diluted 1:100 in Hi-Di formamide (ThermoFisher Scientific, Cat #4311320) and 9 µl of diluted mix was added to 1 µl of PCR product. Samples were mixed and then denatured at 95 °C for 5 minutes before separation on the Genetic Analyzer. For multiplexing where amplicons were generated using individual PCR reactions, 2 µl PCR products from each reaction were pooled, mixed and then 2 µl of the mixture was added to Hi-Di Formamide: ROX mix. The following conditions were used for fragment analysis: Application type: Fragment; Capillary Length: 50 cm; Polymer: POP7; Dye Set: DS30; Filter Set: D; and injection time 15 seconds. Allele sizes were determined using GeneMapper software (ThermoFisher Scientific, Cat # A38888).

### Cloning and sequencing of PCR products

To verify the size of indels, PCR products from the fragment analysis reaction were directly sub-cloned into a pCR4-TOPO vector (ThermoFisher Scientific, Cat # K457502). Plasmid was extracted using a Zymo Plasmid Miniprep kit (Zymo Research, Cat # D4054), and 100 ng DNA from individual clones sequenced using a BigDye Terminator Cycle Sequencing kit (ThermoFisher Scientific, Cat #4337456). The resulting DNA fragments were purified and sequenced using ABI Genetic Analyzer 3500Xl (Applied Biosystems), and aligned with wild type (reference) sequences using SnapGene software (GSL Biotech LLC).

## Results

**Figure 1:**
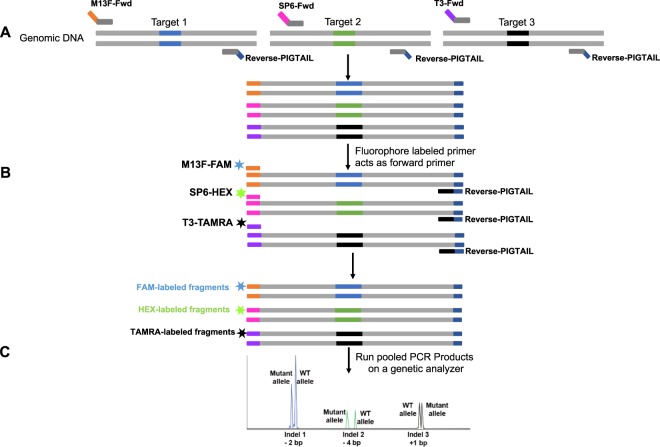
Overview of MultiFRAGing method. (**A**) Primer design strategy for multiple targets. Gene-specific primers are designed to generate 180–300 bp fragments. The gene-specific forward primers are attached with an adapter sequence (M13Fwd, SP6, and T3), and the reverse primer contains a short PIGTAIL sequence to suppress the stutter peaks. Tailed gene-specific primers add M13Fwd or SP6 or T3 adapter sequences in the first few PCR cycles. (**B**) A third primer with same adapter sequence attached to a fluorescent dye (FAM, HEX and TAMRA) is used to generated fluorescently labeled fragments. After few PCR cycles, fluorophore-labeled primers act as forward primer and bind to the respective adapter sequences. In the subsequent PCR cycles, most fragments will incorporate fluorophore thus generating double-stranded fluorescent fragments. Multiple fragments are generated in a single PCR reaction. These fragments can either be tagged with same dye and generate products of different size or tagged with different dyes. (**C**) Pooled PCR products are mixed with a size standard to run on a genetic analyzer. Fragments sizes are plotted, and indel size can be measured based on the expected size of the wild type fragment. Wild-type samples will have one size (allele), while heterozygous samples will show two sizes (alleles).

Our aim was to establish a reliable multiplex method to identify indels from multiple targets in a single PCR reaction that would save time, cost, and increase genotyping efficiency (Fig. 1). The fragment analysis workflow presented here involves labeling fragments with fluorescent dyes to allow multiple colors of fluorescent dyes to be detected in a single sample. ABI genetic analyzers can accommodate at least five different fluorescent dyes (one of which is reserved for a size standard). We used the DS-30 dye set with 6-FAM (blue), Hex (green), NED (yellow), and Rox (Red) (ThermoFisher Scientific, Cat # 4345827). Rox was used as the labeled size standard, leaving the three other colors available. (If more colors are needed, the DS-33 dye set - which contains 6-FAM (Blue), VIC (Green), NED(Yellow), PET (Red) and Liz (Orange) - can be used.) As described in the methods section, gene-specific forward primers were tailed with an adapter sequence (in this case M13Fwd, T3, and SP6 sequences, though any sequence that lacks similarity to the genome could be used). The same sequences were used for dye-labeling. We replaced the NED dye with TAMRA because it uses same color (yellow), is readily available and inexpensive to synthesize.

To establish this method, we used a mutant line carrying mutations at five distinct sites in four different genes (*dfnb31a T1, dfnb31b T1, grhl2a T1, grhl2a T2, and grhl2b T1*). Summary of mutant alleles is listed in Table 2, and detailed data is available in Supplementary Table 1. We tested following combination of fragments based on two parameters: fragments labelled with two or three colors, fragments of two or three sizes, and fragments with different sizes and colors together. First, we genotyped these alleles separately and confirmed the five independent alleles listed in Table 2. These alleles were then used to develop the multiplex method retrospectively. The fluorescent PCR products were combined with a size standard, and run on capillary electrophoresis to identify indels.

**Table 2:** Summary of wild type and mutant alleles, and predicted indel sizes.

Gene	Wild type allele size	Mutant Allele size	Indel size
*dfnb31a*	205.0	203.25	2 bp del
*dfnb31b*	300.46	287.39	13 bp del
*grhl2a*	268.58	267.77	1 bp del
*grhl2a*	268.74	258.61	10 bp del
*grhl2b*	232.14	236.19	4 bp ins

### Amplification of multiple fragments separated by color, and multiplex genotyping in a single reaction

**Figure 2:**
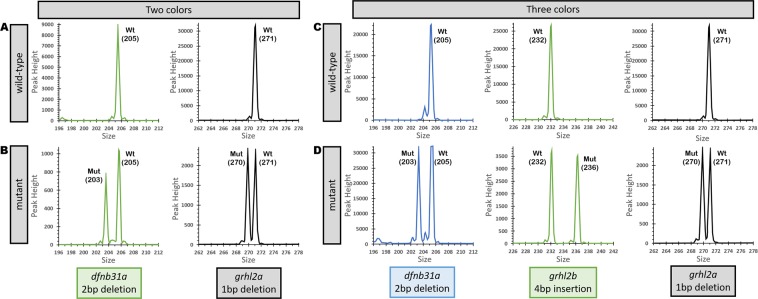
Fragment Analysis PCR plots from wild-type samples and a pool of samples amplified together. (**A**) Wild-type controls for size comparison with two targets separated by different dyes. (**B**) Two targets are separated based on different dyes, showing a 2 bp or 1 bp deletion when compared to wild-type sizes. (**C**) Wild-type controls for size comparison with three targets separated by different dyes. (**D**) Three targets are separated by dye color, and show a 2 bp deletion, 4 bp insertion, and 1 bp deletion compared to wild-type sizes.

We tested two different strategies to multiplex: amplicons separated by size and/or colors. It has been shown that the majority of indels induced by CRISPR/Cas9 are less than 20 bp in size, which makes it possible to design specific primers to generate fragments of different sizes (within a 180–300 bp range), thereby allowing us to vary both fragment length and dye color (Table 1). To test this approach, we first amplified two fragments using two gene-specific primer sets, together with unique third primers linked to different fluorophores (TAMRA and HEX dyes) simultaneously. The resulting amplicons were subjected to fragment analysis in a single reaction. Genotyping showed that control samples (wild-type) generated single peaks (corresponding to the size of the amplicon) for each allele (Fig. 2A), and heterozygous samples generated two peaks (mutant and wild-type). In both cases the second peak was smaller than the wild-type control indicating deletions (2 bp and 1 bp) (Table 2). After testing two fragments by simultaneous amplification and genotyping, we tested three different fragments separated by three unique fluorophores- FAM (Blue), HEX (Green) and TAMRA (Yellow), and genotyped them simultaneously in a single reaction. As expected, three different alleles [corresponding to a 2 bp deletion (*dfnb31a*), 4 bp insertion (*grhl2b*), and 1 bp deletion (*grhl2a*)] were identified successfully (Fig. 2D); the size of the mutant allele was determined by size comparison with control peaks (Fig. 2C).

### Amplification of multiple fragments separated by size, and multiplex genotyping in a single reaction

We hypothesized that given most indels are less than 20 bp, multiple targets can be separated and identified by size. The choice of size is dependent on the fragment analysis size standard; we use 400 HD ROX, which is designed for sizing DNA fragments in the 50–400 nucleotide range. We normally design primers to generate fragments between 180–300 bp. For other size standards (e.g. 500 HD ROX, or 500 LIZ), amplicons of up to 500 bp can be generated. We tested this strategy by amplifying two, three, and four targets of different sizes simultaneously in a single reaction. Two and three targets amplified successfully, however, four target amplification was not successful. We tested different DNA polymerases (Platinum Taq, AmpliTaq Gold, and QIAGEN HotStarTaq), including some specialized for multiplex PCR (NEB Multiplex PCR 5X Master Mix, and Phusion Multiplex PCR Master Mix). Surprisingly, standard polymerases were more effective than multiplex PCR master mixes: Qiagen HotStarTaq, and Platinum Taq performed slightly better than AmpliTaq Gold. We successfully amplified three targets of 205 bp, 232 bp, and 269 bp simultaneously (Supplementary Fig. 1). When the collection of amplified fragments was genotyped, each of the two and three mutant alleles were successfully identified (Supplementary Fig. 2B,D respectively). The control sizes are shown in Supplementary Fig. 2A,C. Thus, this strategy of amplifying three targets simultaneously can increase genotyping throughput by three-fold.

### Genotyping of individually amplified fragments in a pool

**Figure 3:**
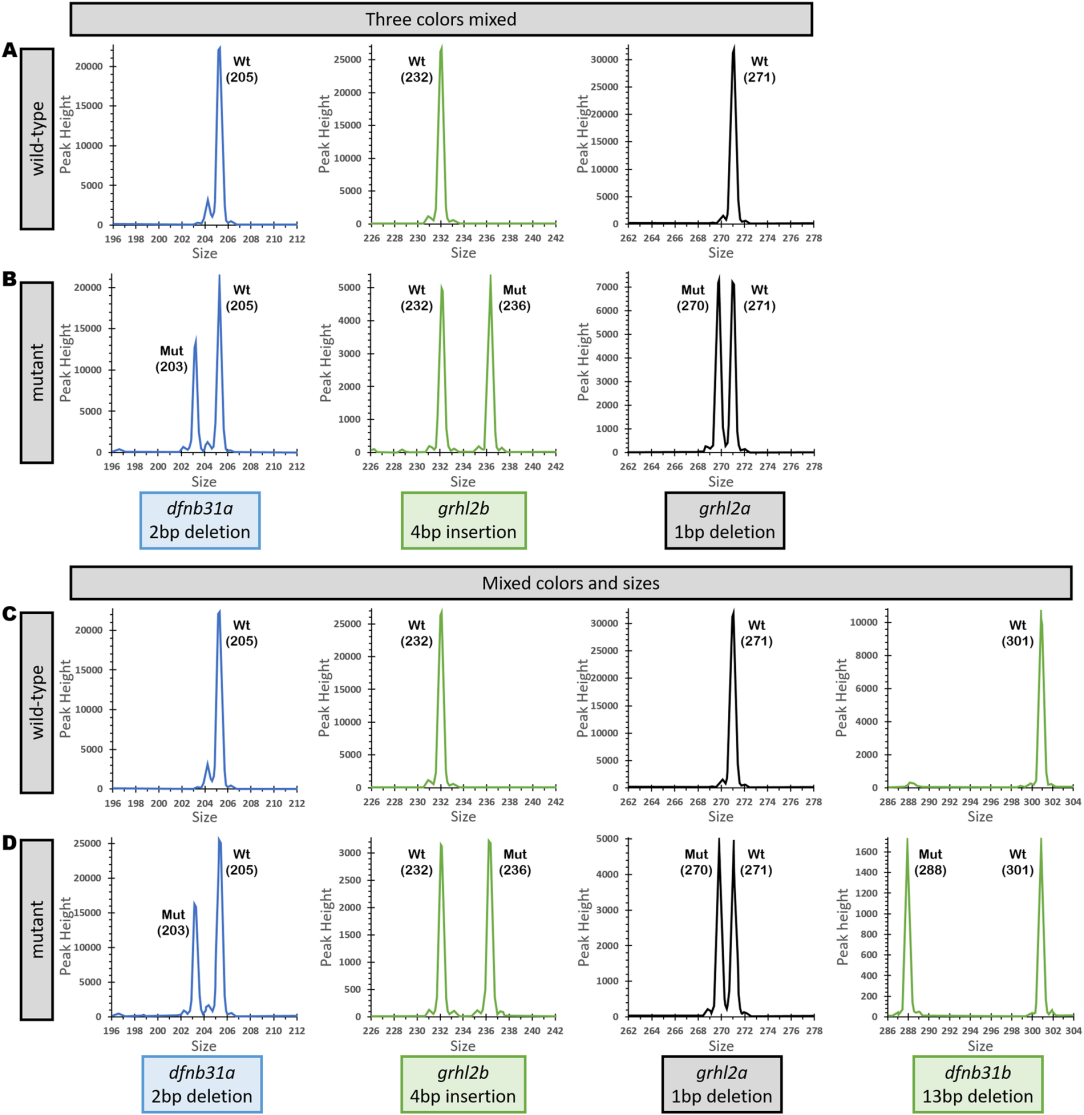
Fragment Analysis plots from wild-type controls and mutants from fragments separated by color, sizes and amplified individually, then pooled together for genetic analyzer. (**A**) Wild-type controls for size comparison with three targets separated by different dyes. (**B)** Three targets separated by different dyes showing indels of 2 bp deletion, 4 bp insertion, and 1 bp deletion when compared to wild-type sizes. (**C)** Wild-type controls for size comparison with targets separated by size and dye color. (**D)** Four targets separated by a combination of different sizes and dye colors showing indels of 2 bp deletion, 4 bp insertion,1 bp deletion, and 13 bp deletion when compared to wild-type sizes.

In many cases only one sgRNA is used to target a single gene, and these targets are amplified individually. We tested whether individually amplified targets with different colors and/or sizes could be pooled together and still allow indels to be identified in a single reaction. We distinguished these targets in multiple ways: by size, color, or a mix of colors and sizes. To test this approach, 3 or 4 different targets were amplified individually in separate reactions. We combined 2 µl of three or four different PCR products, and 2 µl of pooled product was run on ABI genetic analyzer for genotyping in a single well. First, we mixed fragments amplified from three targets separated by three colors. In a second pool, a combination of sizes (203, 232, 271, and 301 bp) and colors (fragments of 232 and 301 bp have the HEX fluorophore, while 203 uses FAM, and 271 uses TAMRA fluorophore) was tested. In both cases, all alleles were identified successfully and accurately as shown in Fig. 3C,D. Furthermore, pooling three and four fragments of different sizes and genotyping them in a single reaction yielded the same results: all mixed alleles were identified successfully (Supplementary Fig. 3B,D). Wild-type sizes are shown in Supplementary Fig. 3A,B. These results demonstrate that it is possible to pool multiple PCR products to increase the genotyping throughput up to four-fold, and save the cost of consumables time, and labor.

### Verification of mutant alleles by sanger sequencing

**Figure 4:**
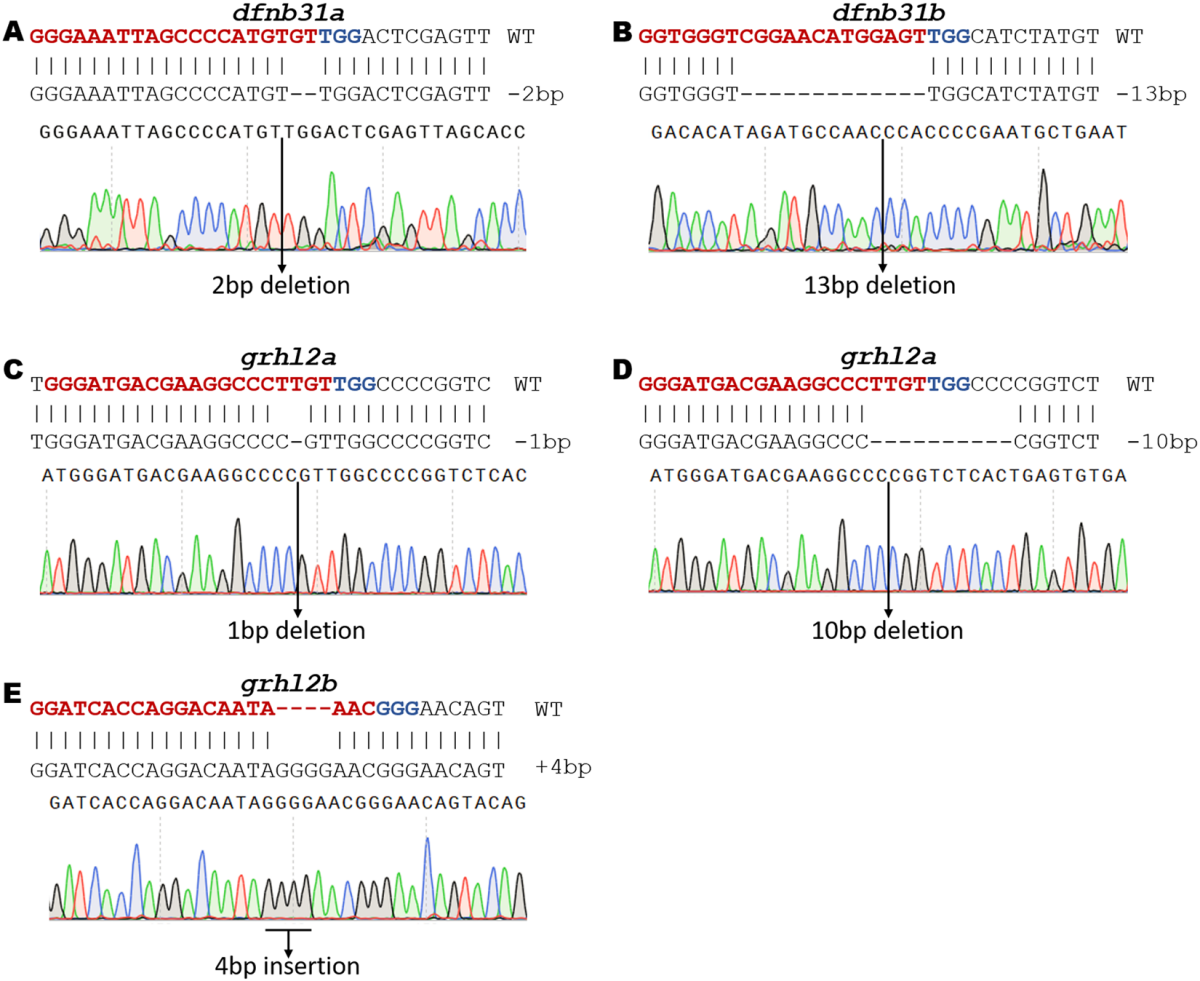
Validation of indels by Sanger Sequencing. Each indel that was identified by fragment analysis was sequenced using the Sanger method to establish the correlation between fragment analysis and Sanger sequencing Data. All indels from fragment analysis showed similar indel size in Sanger sequencing. (**A**) Alignment and chromatogram confirming a 2 bp deletion in *dfnb31a*. (**B)** Alignment and chromatogram confirming a 13 bp deletion in *dfnb31b*. (**C)** Alignment and chromatogram confirming a 1 bp deletion in *grhl2a*. (**D)** Alignment and chromatogram confirming a 10 bp deletion in *grhl2a*. (**E)** Alignment and chromatogram confirming a 4 bp insertion in *grhl2b*.

While this fragment analysis-based genotyping provides the size of the indels, it does not provide the exact sequences of mutations. In order to do this, amplicons must be sequenced the first time mutants are identified (which we normally do to validate the fragment analysis data). Here, we confirmed all alleles tested to demonstrate the feasibility of multiplex method. We genotyped five different alleles using fluorescent PCR: 2 bp deletion in *dfnb31a;* 13 bp deletion *dfnb31b; 1 bp* insertion and 10 bp deletion in *grhl2a; 4 bp* deletion in *grhl2b* (Fig. 4A–E). Amplicons from heterozygous samples were sub-cloned in a TOPO vector, and DNA from individual clones was sequenced. Sanger sequencing confirmed all five alleles as identified from fluorescent PCR. Heterozygous samples can also be sequenced directly, and sequencing data can be analyzed by Poly Peak Parser (http://yosttools.genetics.utah.edu/PolyPeakParser/), a tool to parse double peaks from Sanger sequencing chromatograms into wild-type and alternative alleles (Supplementary Fig. 4A,B)^25^.

## Discussion

Emerging genome editing technologies such as CRISPR/Cas9, and TALENs have revolutionized targeted mutagenesis in zebrafish. While they allow gene knockouts to be generated in a high-throughput manner, the identification of mutated alleles at a similar rate and in a cost-effective manner has been lacking. Several strategies are being used such as DNA sequencing, fluorescent PCR based fragment analysis, T7 endonuclease assay, PAGE-based genotyping, high-resolution melting analysis (HRMA), and melting curve analysis^18^. Each of these techniques has their own strengths and weaknesses: the most sensitive method of identifying allele is Sanger sequencing but it is expensive, time-consuming, and low throughput; HRM, melting curve analysis, and fluorescent PCR methods are high-throughput; the HRM and melting curve analysis methods are low cost, but they lack sensitivity due to the high number of polymorphisms in zebrafish. The fluorescent PCR based fragment analysis method is high-throughput and highly sensitive, but also high cost. The fragment analysis method was first used to identify indels generated by ZFNs in zebrafish^20^, and later modified to include a third fluorescent primer (to reduce cost) and a PIGTAIL (to suppress stutter peaks^22^). This method was used to identify indels generated by CRISPR/Cas9 in a high-throughput manner^9,10^, and modified to develop CRISPR-STAT (CRISPR-Somatic Tissue Activity Test), an assay to determine the sgRNA activity *in vivo*^21^. In order to further reduce cost and increase throughput, we adapted this method to identify multiple alleles in a single reaction, showing that fragment analysis can be used to genotype multiple alleles simultaneously. The result is a more robust and cost-effective genotyping method. The cost of this method is approximately $2–3 per reaction in our lab. All alleles identified by fragment analysis were confirmed by sequencing, and the sequencing data was co-related with the fragment analysis data, which confirms the sensitivity of this method.

Multiplexing can be done using two different approaches: amplification of up to three targets in a single PCR reaction, followed by genotyping of the products in a single reaction; another approach is to amplify targets individually and pool the products for genotyping in a single reaction, which allows us to genotype up to four targets. Another way to genotype four targets simultaneously is to amplify two targets simultaneously in a single reaction, and then pool fragments from two reactions. While we have demonstrated the use of multiplexing in zebrafish, this method can be easily used in other systems.

## Supplementary information

Supplementary Information.
