# Supplementary material for: MultiFRAGing: Rapid and Simultaneous Genotyping of Multiple Alleles in a Single Reaction
Source: Sci Rep. 2020 Feb 21;10:3172. doi: 10.1038/s41598-020-59986-1 (PMC7035419; doi:10.1038/s41598-020-59986-1)

Supplementary Information

MultiFRAGing: Rapid and Simultaneous Genotyping of Multiple Alleles in a Single Reaction

Cassidy Petree[1], Gaurav K. Varshney[1,*]

Supplementary Figure 1

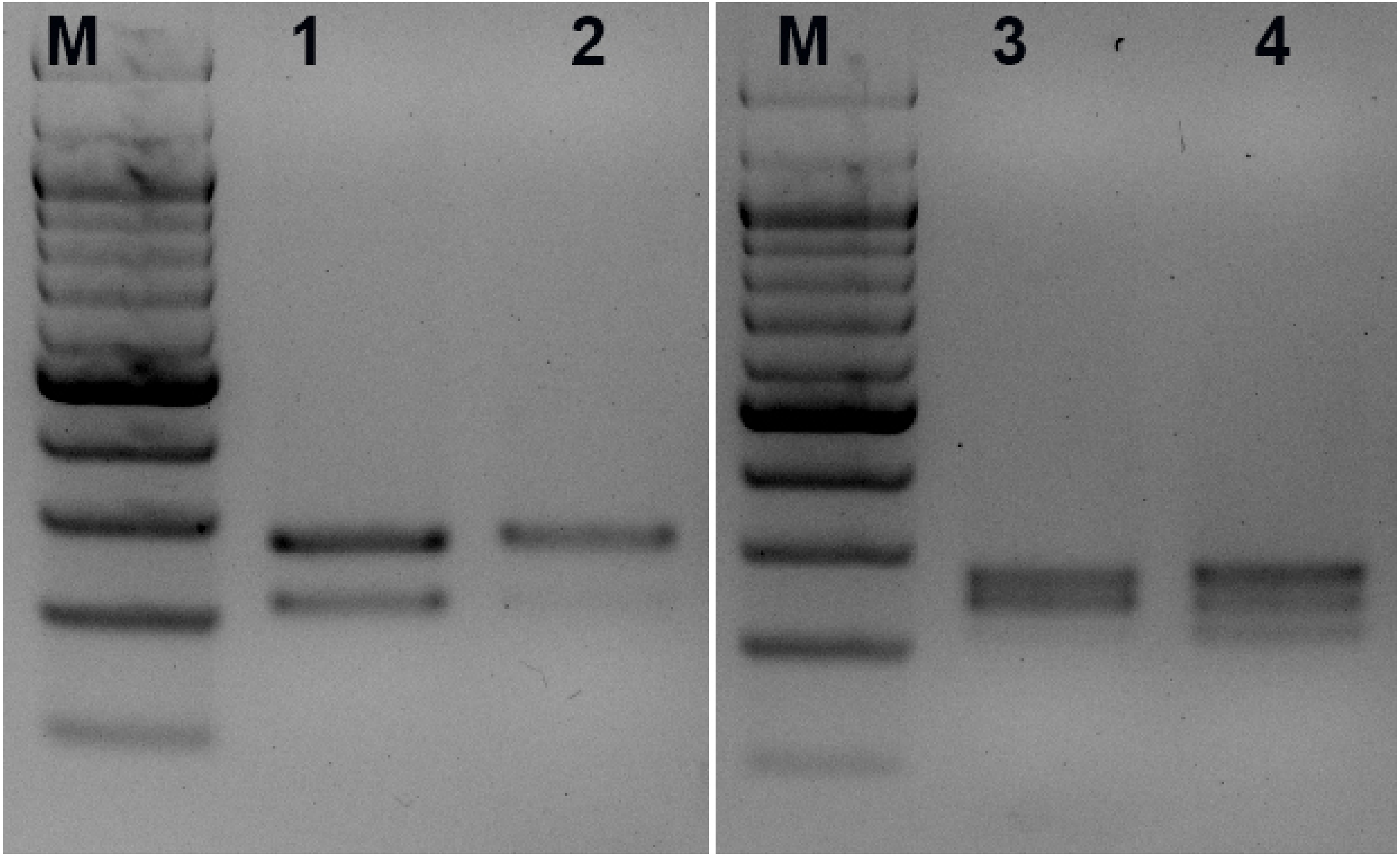

Supplementary Figure 2

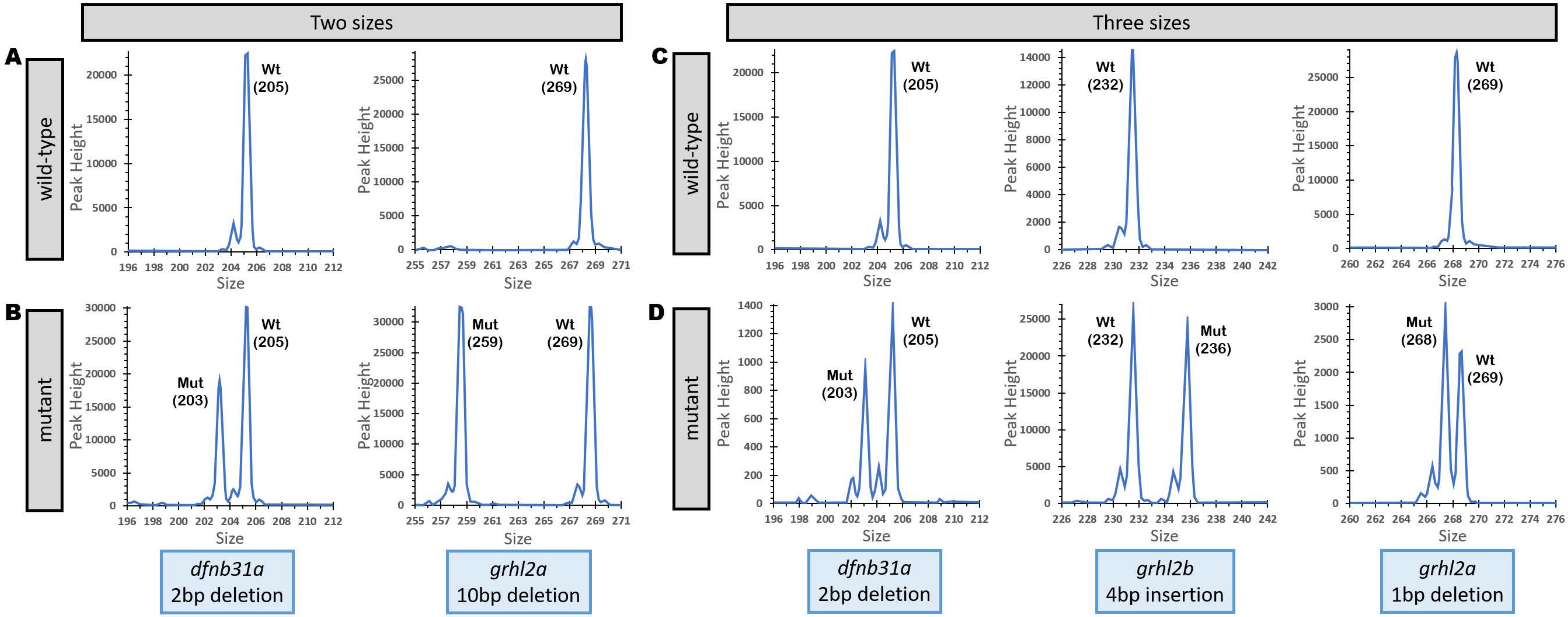

Supplementary Figure 3

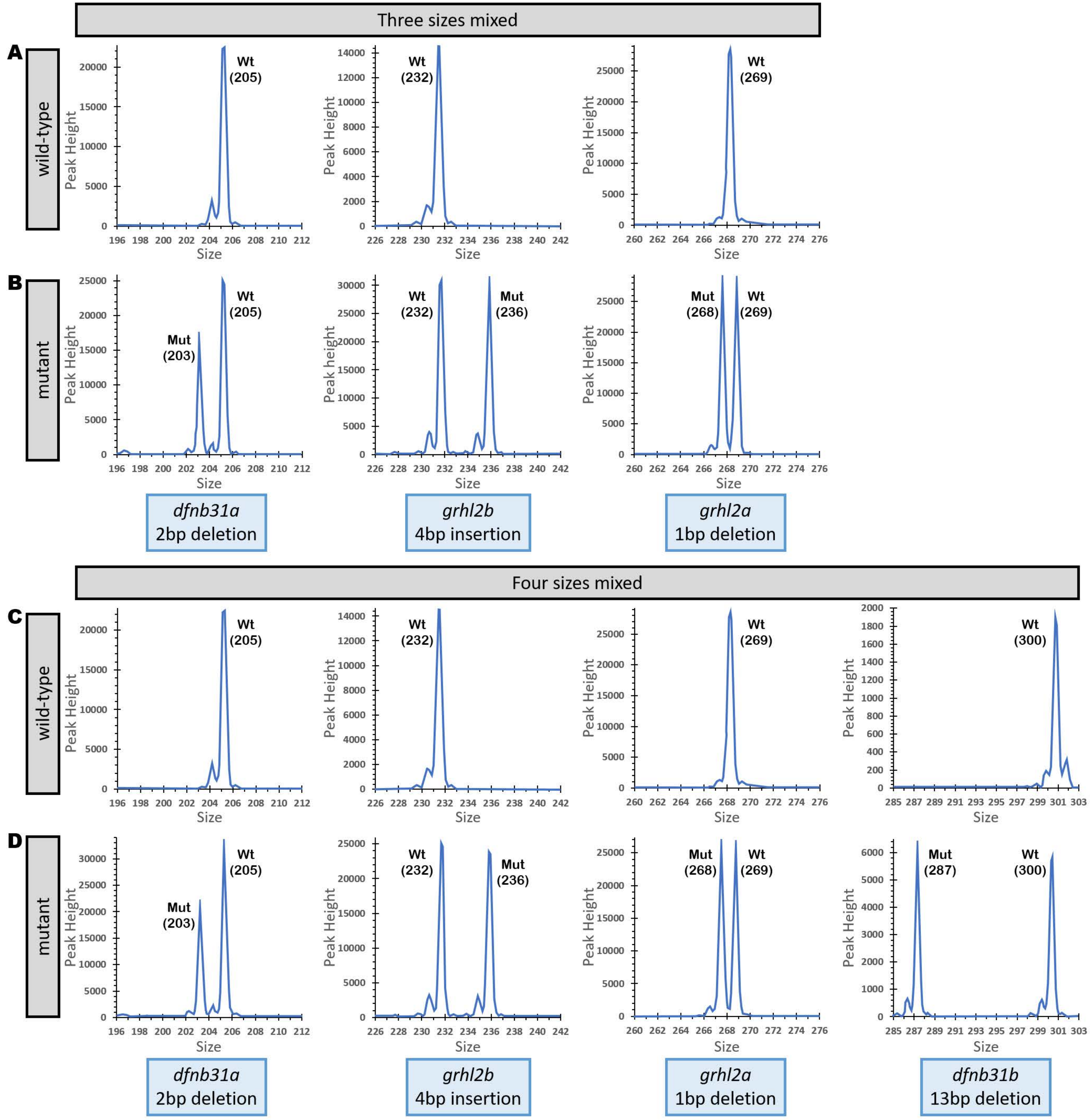

Supplementary Figure 4

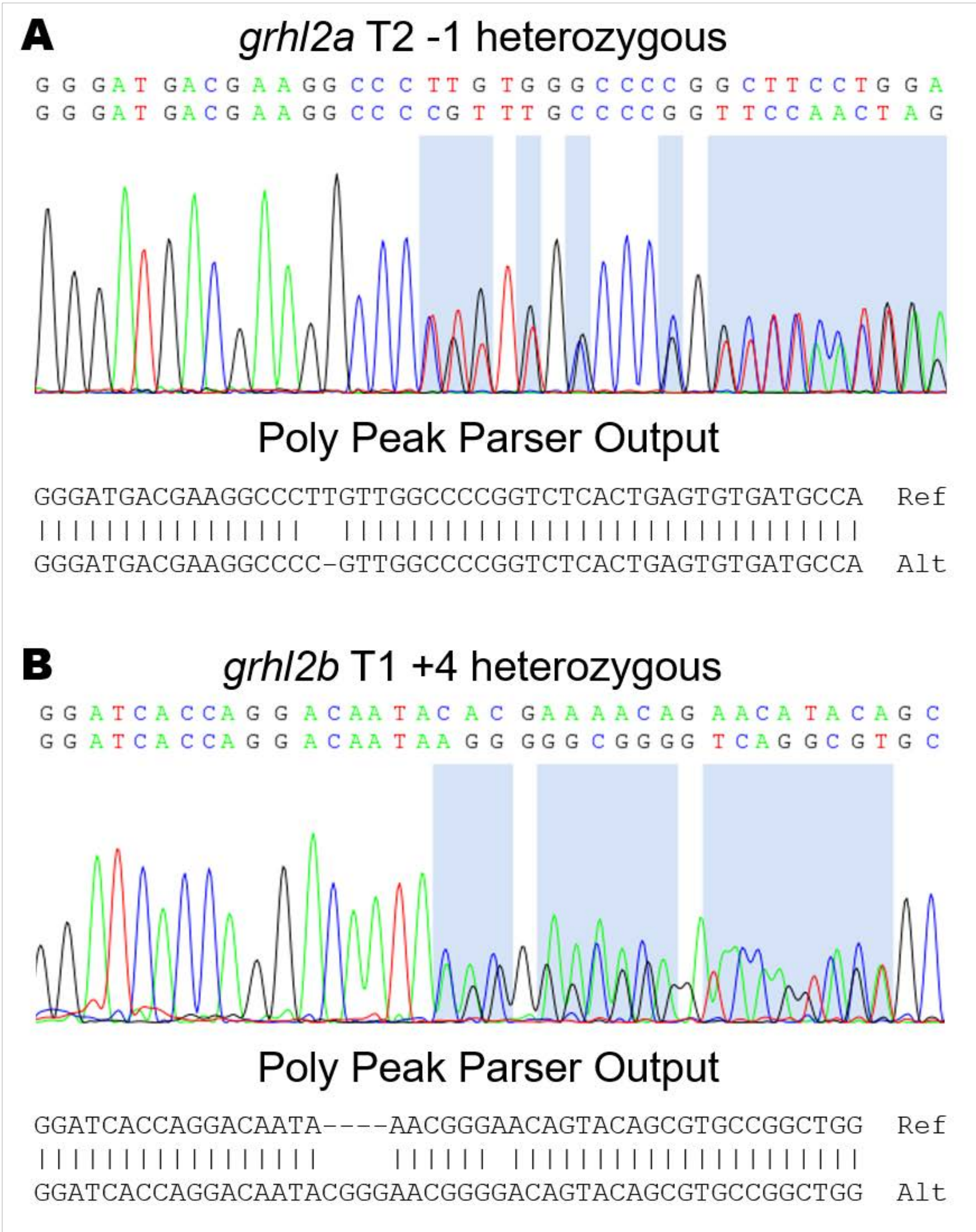

Supplementary Figure 1: Separation of PCR products from pooled PCR on agarose gel electrophoresis. M: 100bp marker, Lane 1: Two fragments with different sizes, same color. Lane 2: Two different fragments with different sizes, different color. Lane 3: Three different fragments with different sizes, same color. Lane 2: Three different *fragments* with different sizes, different color. Original Gel Image data available as supplementary file. Gel image was modified only for presentation purpose but no part of the gel was modified to change the results.

Supplementary Figure 2: Fragment Analysis PCR plots from wild-type samples and a pool of samples amplified together. **A).** Wild-type controls for size comparison with two targets separated by different sizes. **B)** Two targets were separated based on size, and show a 2bp and 10bp deletion when compared to wild-type sizes. **C)** Wild-type controls for size comparison with three targets separated by different sizes. **D)** Three targets are separated based on size and show a 2bp deletion, 4bp insertion, and 1bp deletion when compared to wild-type sizes.

Supplementary Figure 3. Fragment Analysis PCR plots from wild-type controls, and mutants from fragment separated by sizes and generated by PCR individually, and pooled together for genetic analyzer. **A**) Wild-type controls for size comparison with three targets separated by different sizes. **B)** Three targets separated by different sizes showing indels of 2bp deletion, 4bp insertion, and 1bp deletion when

compared to wild-type sizes. **C)** Wild-type controls for size comparison with four targets separated by different sizes. **D)** Four targets separated by different sizes showing indels of 2 bp deletion, 4bp insertion,1bp deletion, and 13bp deletion when compared to wild-type sizes.

Supplementary Figure 4: Chromatograms and output from Poly Peak Parser for heterozygous samples. **A).** Chromatogram and output for *grhl2a* showing the same 1bp deletion as Sanger sequencing of homozygous samples. **B)** Chromatogram and output for *grhl2b* showing the same 4bp insertion as Sanger sequencing of homozygous samples.

Supplementary Table 1. Summary of ABI Genetic Analyzer Output

	dfnb31a T1-FAM				*grhl2a* T1-FAM			
Sample Name	Allele Size 1	Allele Size 2	Peak Height 1	Peak Height 2	Allele Size 1	Allele Size 2	Peak Height 1	Peak Height 2
B01	203.18	205.19	19391	30248	258.47	268.69	32449	32643
G01	203.25	205	32148	31952	267.77	268.58	32548	32548
H01	203.1	205.39	32374	32374	258.52	268.63	32521	32747
	dfnb31a T1-HEX				*grhl2a* T1-TAMRA			
Sample Name	Allele Size 1	Allele Size 2	Peak Height 1	Peak Height 2	Allele Size 1	Allele Size 2	Peak Height 1	Peak Height 2
A01	203.54	205.59	764	1072	269.83	271.06	4524	4376
B01	203.56	205.62	534	819	260.91	271.03	4236	3923

	dfnb31a T1-FAM				*grhl2b* T1-FAM				*grhl2a* T1-FAM			
Sample Name	Allele Size 1	Allele Size 2	Peak Height 1	Peak Height 2	Allele Size 1	Allele Size 2	Peak Height 1	Peak Height 2	Allele Size 1	Allele Size 2	Peak Height 1	Peak Height 2
A01	203.07	205.19	1000	1409	231.57	235.72	27077	25137	267.41	268.7	3043	2367
A02	203.11	205.18	12333	17445	231.42	236.07	31667	31822	267.55	268.69	10610	10039
B02	203.15	205.25	12003	18124	231.49	236.04	31092	31281	258.61	268.74	8829	8229
	dfnb31a T1-FAM				*grhl2b* T1-HEX				*grhl2a* T1-TAMRA			
Sample Name	Allele Size 1	Allele Size 2	Peak Height 1	Peak Height 2	Allele Size 1	Allele Size 2	Peak Height 1	Peak Height 2	Allele Size 1	Allele Size 2	Peak Height 1	Peak Height 2
C01	203.24	205.43	30959	32040	232.14	236.31	3875	3618	269.78	271.04	2461	2434
D01	203.13	205.33	26770	31737	232.14	236.19	3398	3317	260.92	270.98	2352	2200

Mixed

	dfnb31a T1-FAM				*grhl2b* T1-FAM				*grhl2a* T1-FAM			
Sample Name	Allele Size 1	Allele Size 2	Peak Height 1	Peak Height 2	Allele Size 1	Allele Size 2	Peak Height 1	Peak Height 2	Allele Size 1	Allele Size 2	Peak Height 1	Peak Height 2
A01	203.18	205.22	17362	25763	231.69	235.78	31911	31161	267.54	268.78	29295	29068
	dfnb31a T1-FAM				*grhl2b* T1-HEX				*grhl2a* T1-TAMRA			
Sample Name	Allele Size 1	Allele Size 2	Peak Height 1	Peak Height 2	Allele Size 1	Allele Size 2	Peak Height 1	Peak Height 2	Allele Size 1	Allele Size 2	Peak Height 1	Peak Height 2
C01	203.2	205.26	13478	21662	232.07	236.33	5165	5418	269.84	270.97	7645	7524

	dfnb31a T1-FAM				*grhl2b* T1-FAM				*grhl2a* T1-FAM			
**Sample Name**	**Allele Size 1**	**Allele Size 2**	**Peak Height 1**	**Peak Height 2**	**Allele Size 1**	**Allele Size 2**	**Peak Height 1**	**Peak Height 2**	**Allele Size 1**	**Allele Size 2**	**Peak Height 1**	**Peak Height 2**
A01	203.22	205.29	13132	20281	231.66	235.83	25840	24513	267.56	268.82	27248	27074

Dfnb31b T1-FAM			
**Allele Size 1**	**Allele Size 2**	**Peak Height 1**	**Peak Height 2**
287.39	300.46	6481	6129

	dfnb31a T1-FAM				*grhl2b* T1-HEX				*grhl2a* T1-TAMRA			
**Sample Name**	**Allele Size 1**	**Allele Size 2**	**Peak Height 1**	**Peak Height 2**	**Allele Size 1**	**Allele Size 2**	**Peak Height 1**	**Peak Height 2**	**Allele Size 1**	**Allele Size 2**	**Peak Height 1**	**Peak Height 2**
C01	203.13	205.21	9540	14890	232.02	236.19	3209	3282	269.84	271.1	5035	4996

Dfnb31b T1-FAM			
**Allele Size 1**	**Allele Size 2**	**Peak Height 1**	**Peak Height 2**
287.82	300.81	1740	1733

Raw Image of DNA gel & Chromatograms from fragment analysis

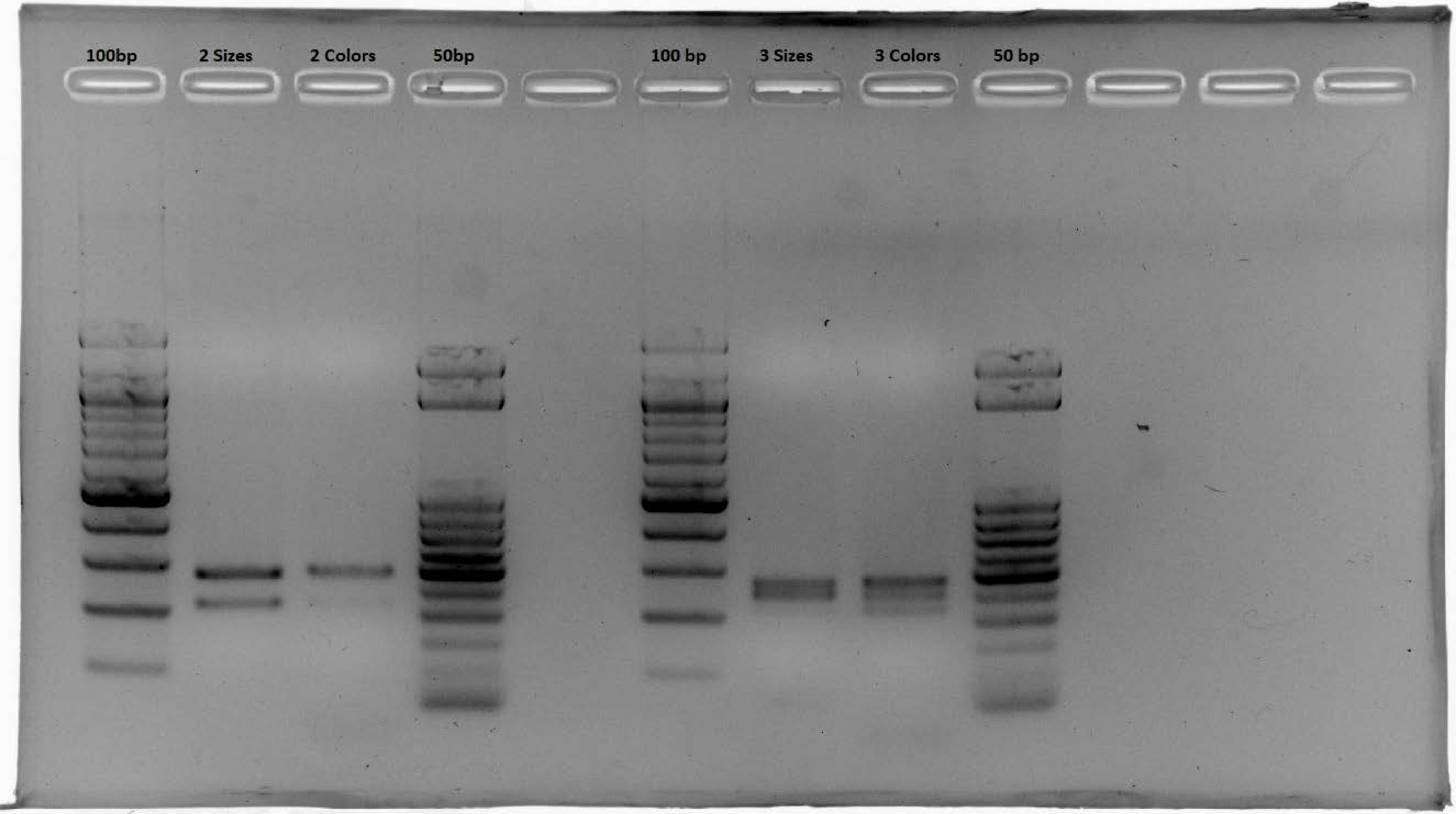

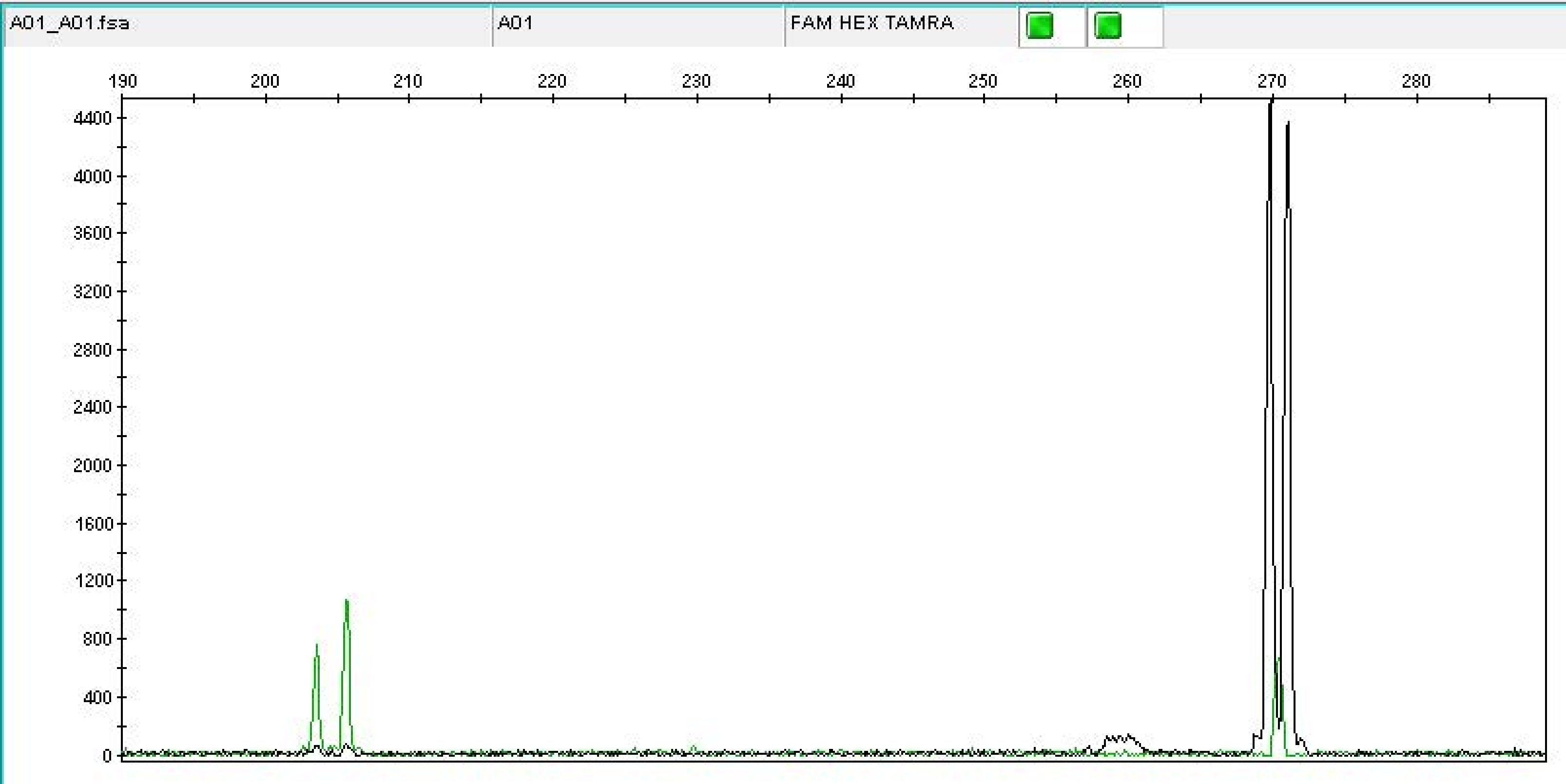
A01_A01.fsa
A01
FAM HEX TAMRA
190 200 210 220 230 240 250 260 270 280
4400 4000 3600 3200 2800 2400 2000 1600 1200 800 400 0

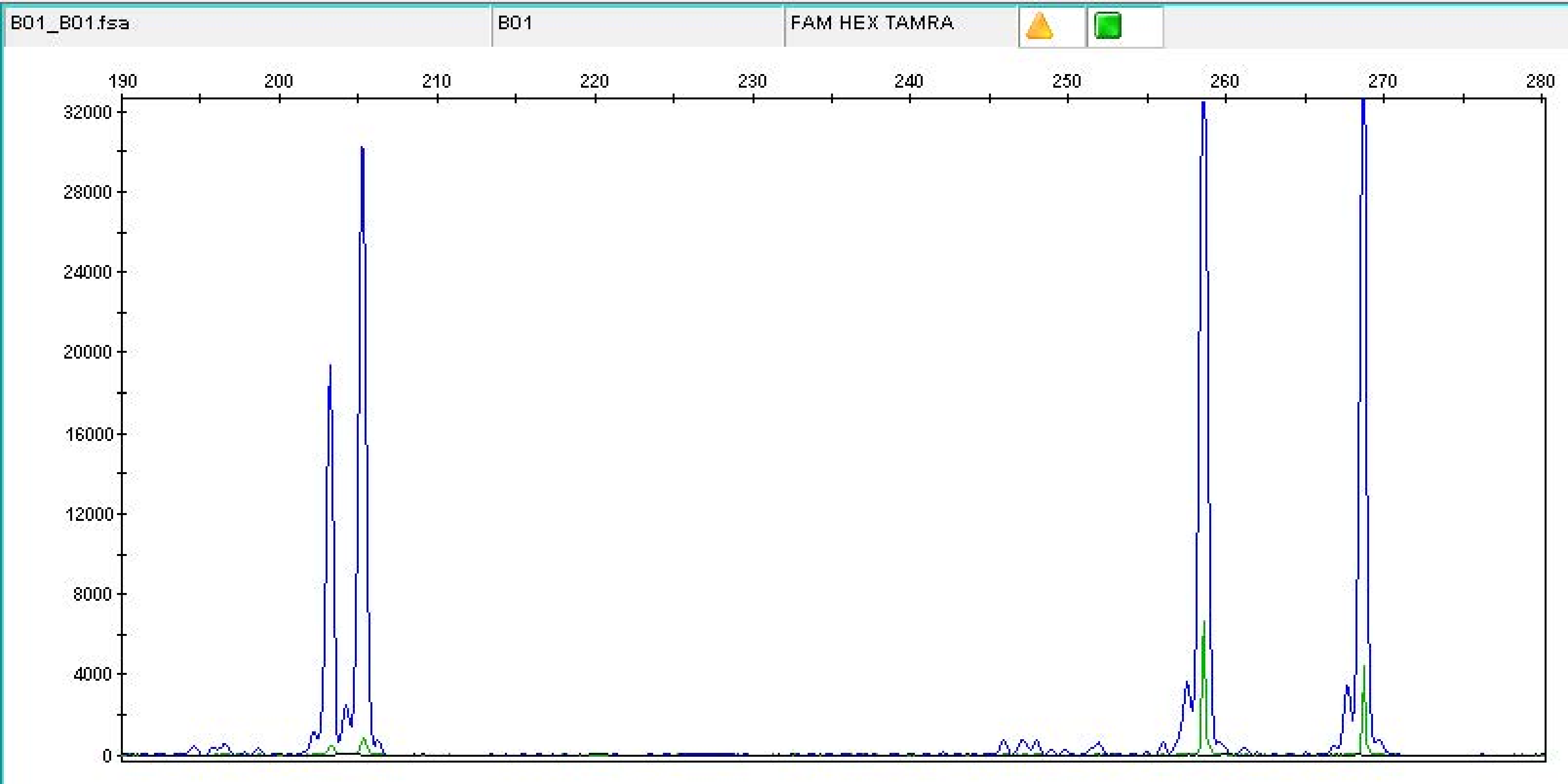
B01_B01.fsa
B01
FAM HEX TAMRA
190
200
210
220
230
240
250
260
270
280
32000
28000
24000
20000
16000
12000
8000
4000
0

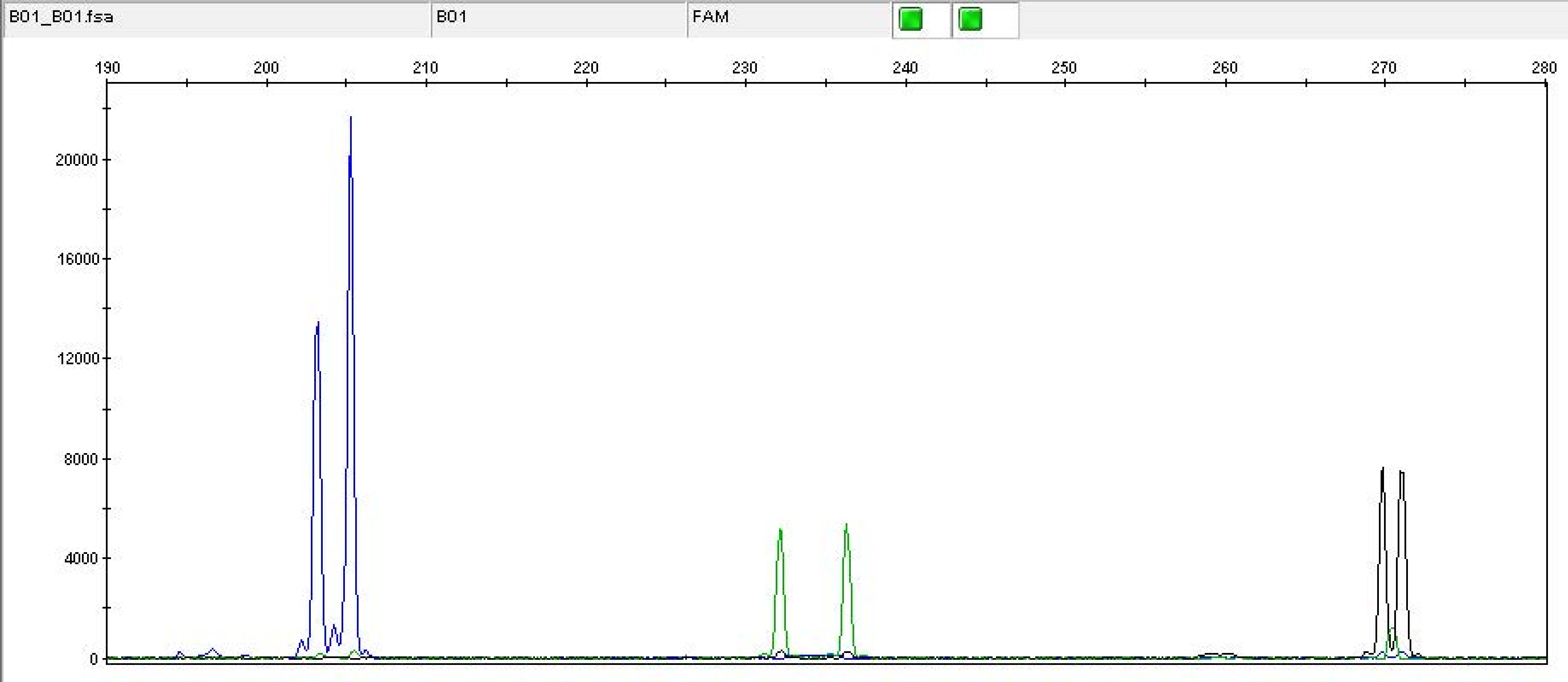

B01_B01.fsa
B01
FAM
190
200
210
220
230
240
250
260
270
280
20000
16000
12000
8000
4000
0

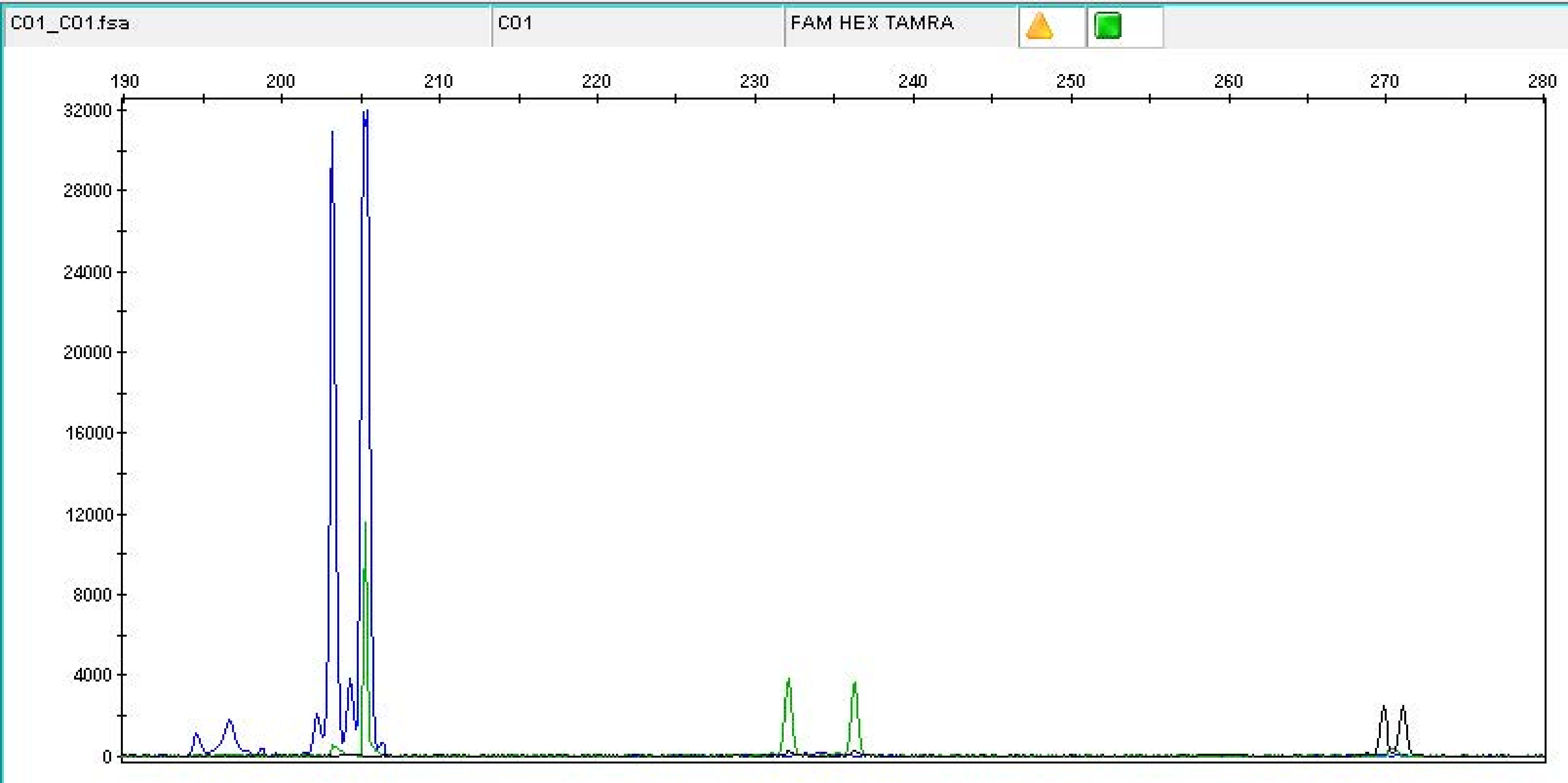
C01_C01.fsa
C01
FAM HEX TAMRA
190
200
210
220
230
240
250
260
270
280
32000
28000
24000
20000
16000
12000
8000
4000
0

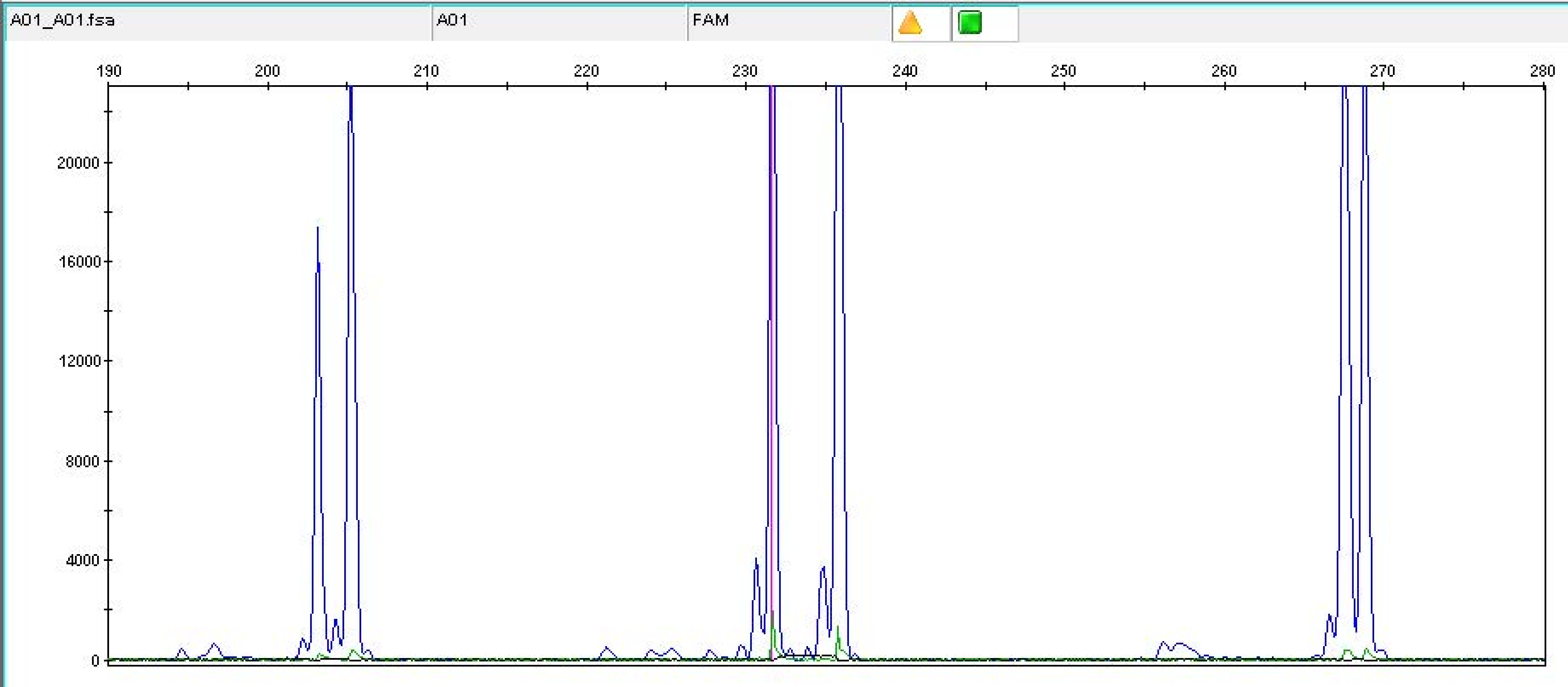
A01_A01.fsa
A01
FAM
190
200
210
220
230
240
250
260
270
280
20000
16000
12000
8000
4000
0

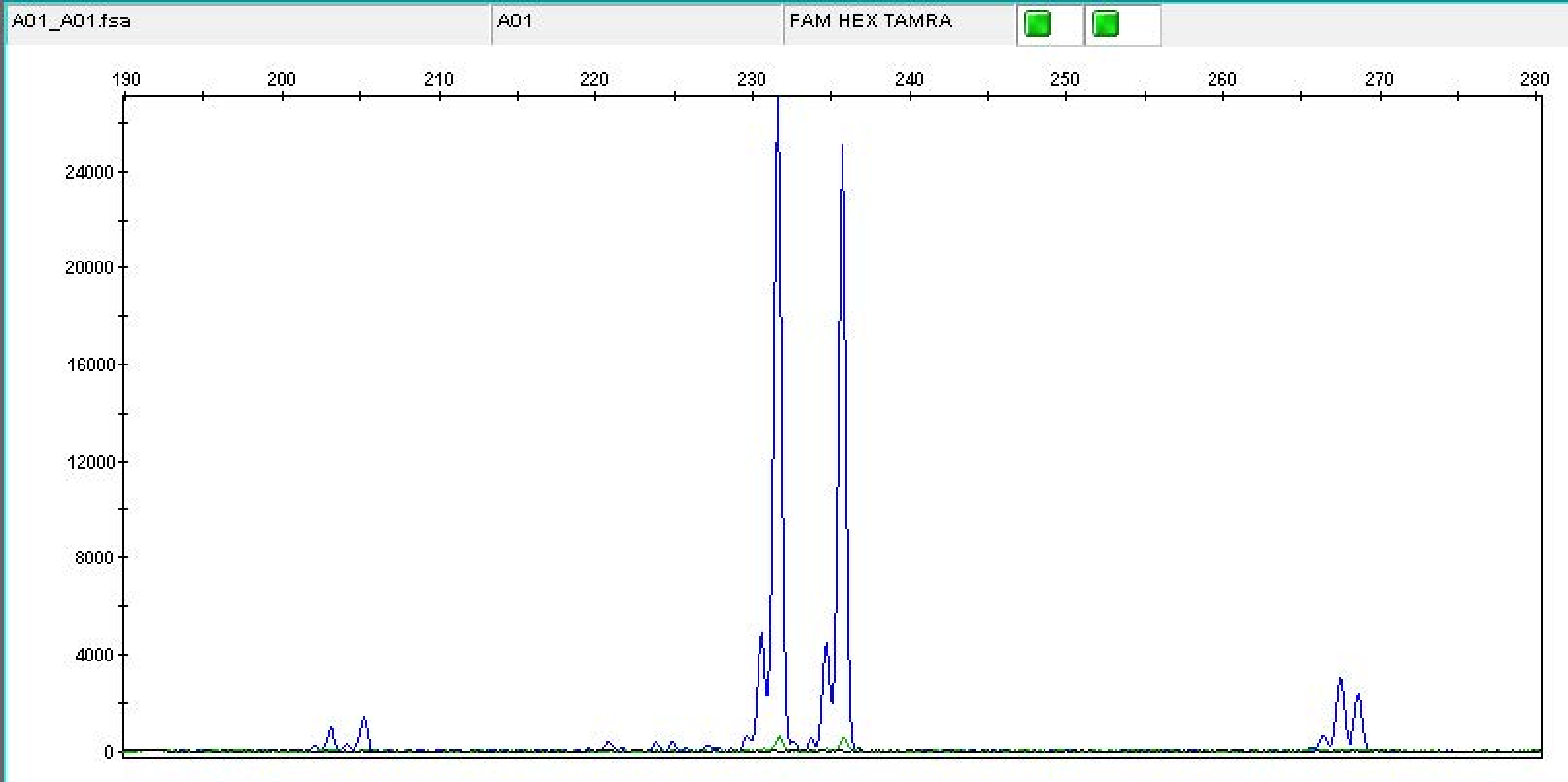
A01_A01.fsa
A01
FAM HEX TAMRA
190
200
210
220
230
240
250
260
270
280
24000
20000
16000
12000
8000
4000
0

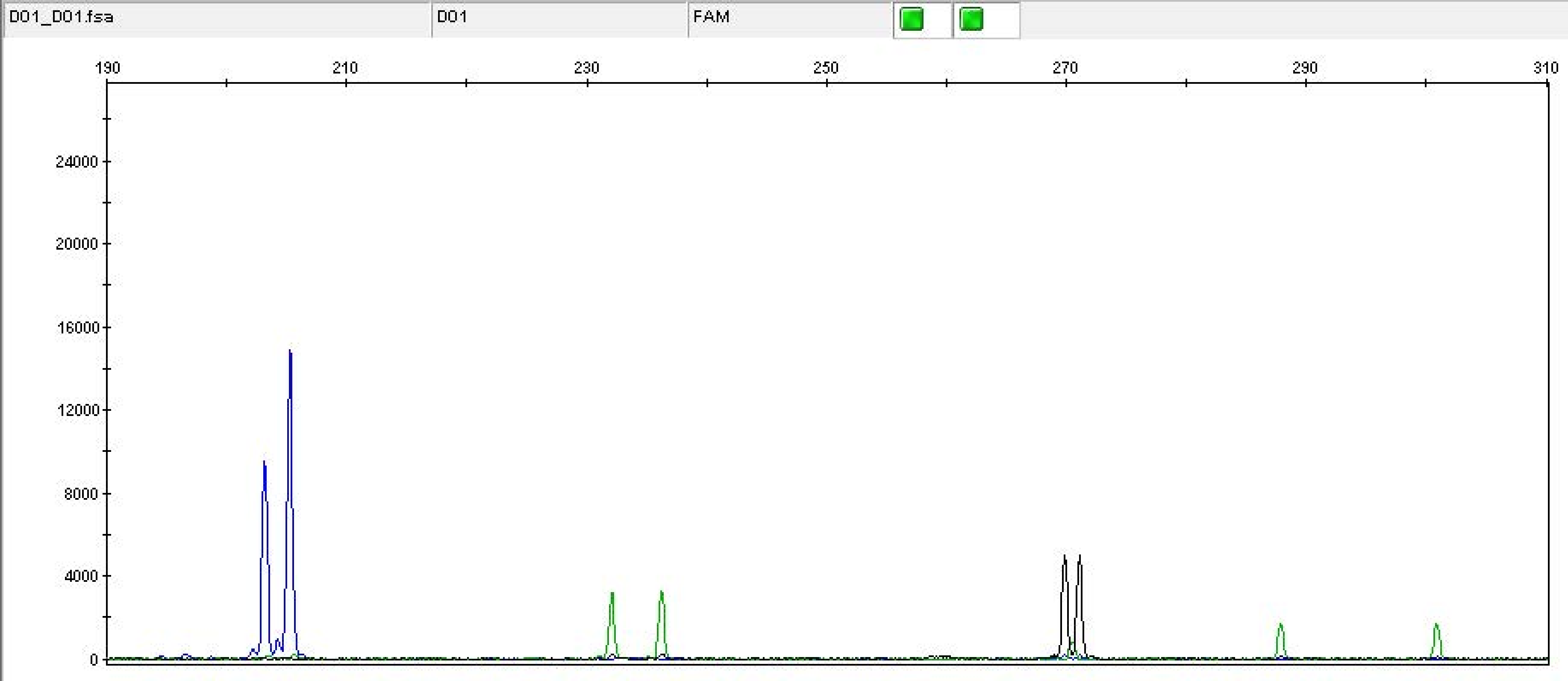
D01_D01.fsa
D01
FAM
190
210
230
250
270
290
310
24000
20000
16000
12000
8000
4000
0

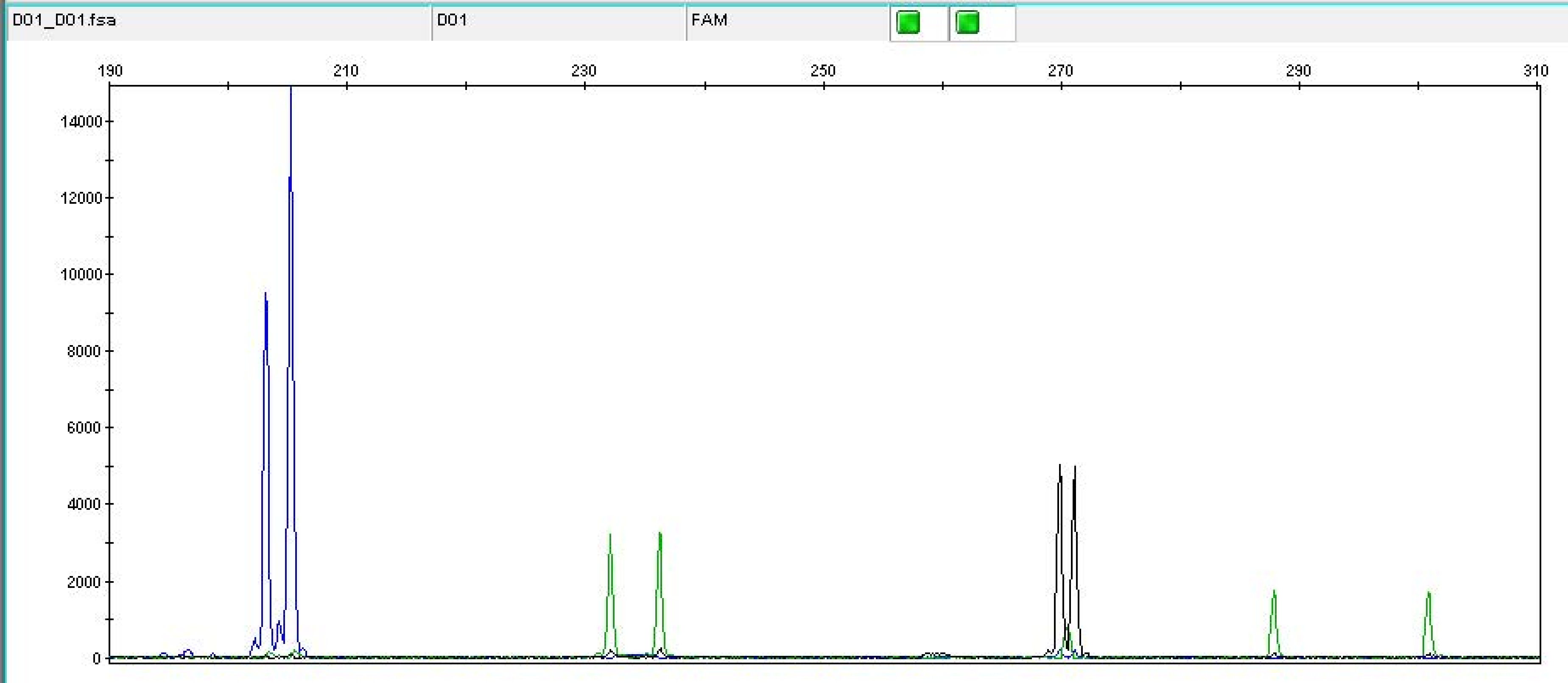
D01_D01.fsa
D01
FAM
190
210
230
250
270
290
310
14000
12000
10000
8000
6000
4000
2000
0

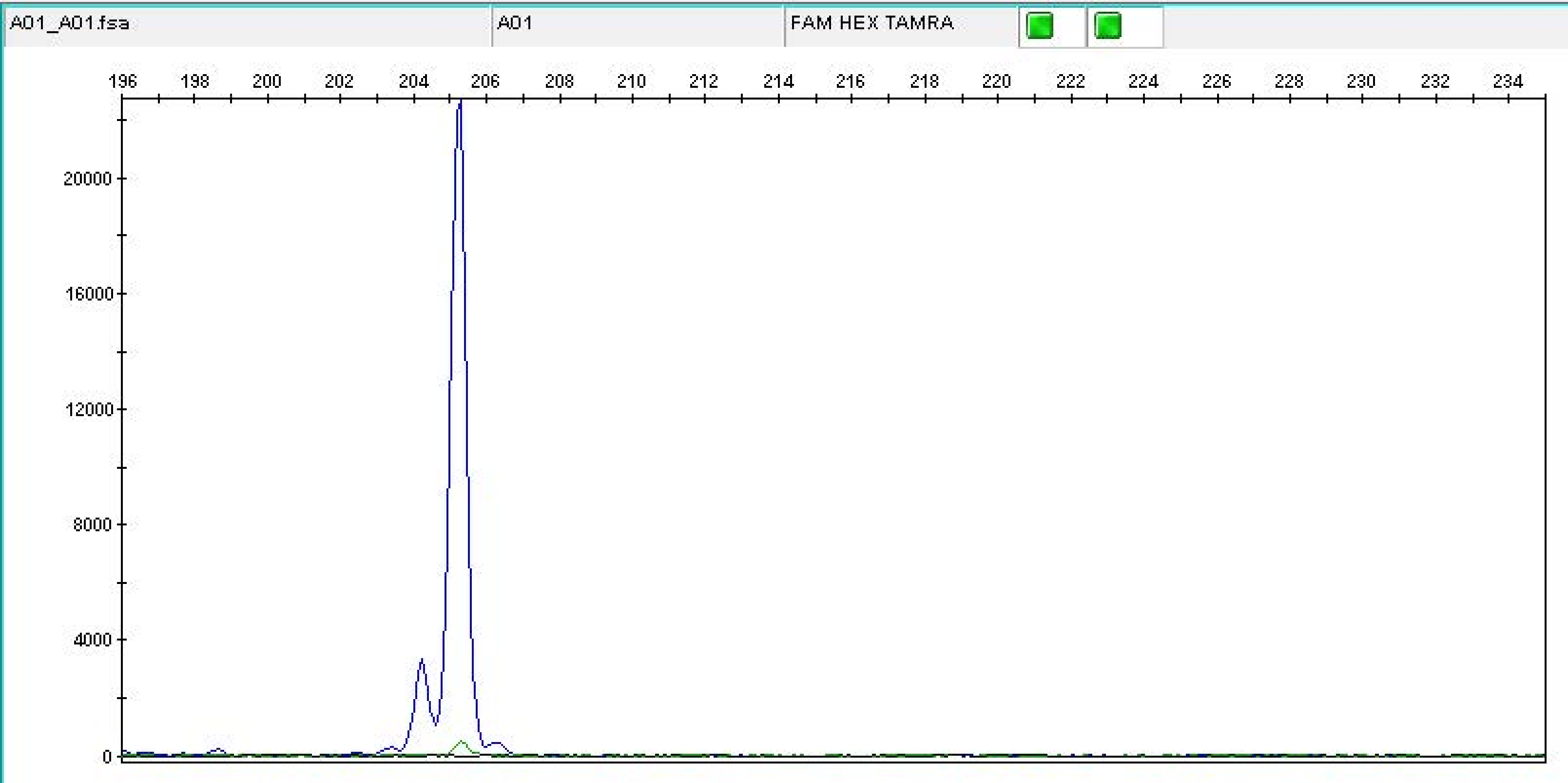
A01_A01.fsa
A01
FAM HEX TAMRA
196 198 200 202 204 206 208 210 212 214 216 218 220 222 224 226 228 230 232 234
20000
16000
12000
8000
4000
0

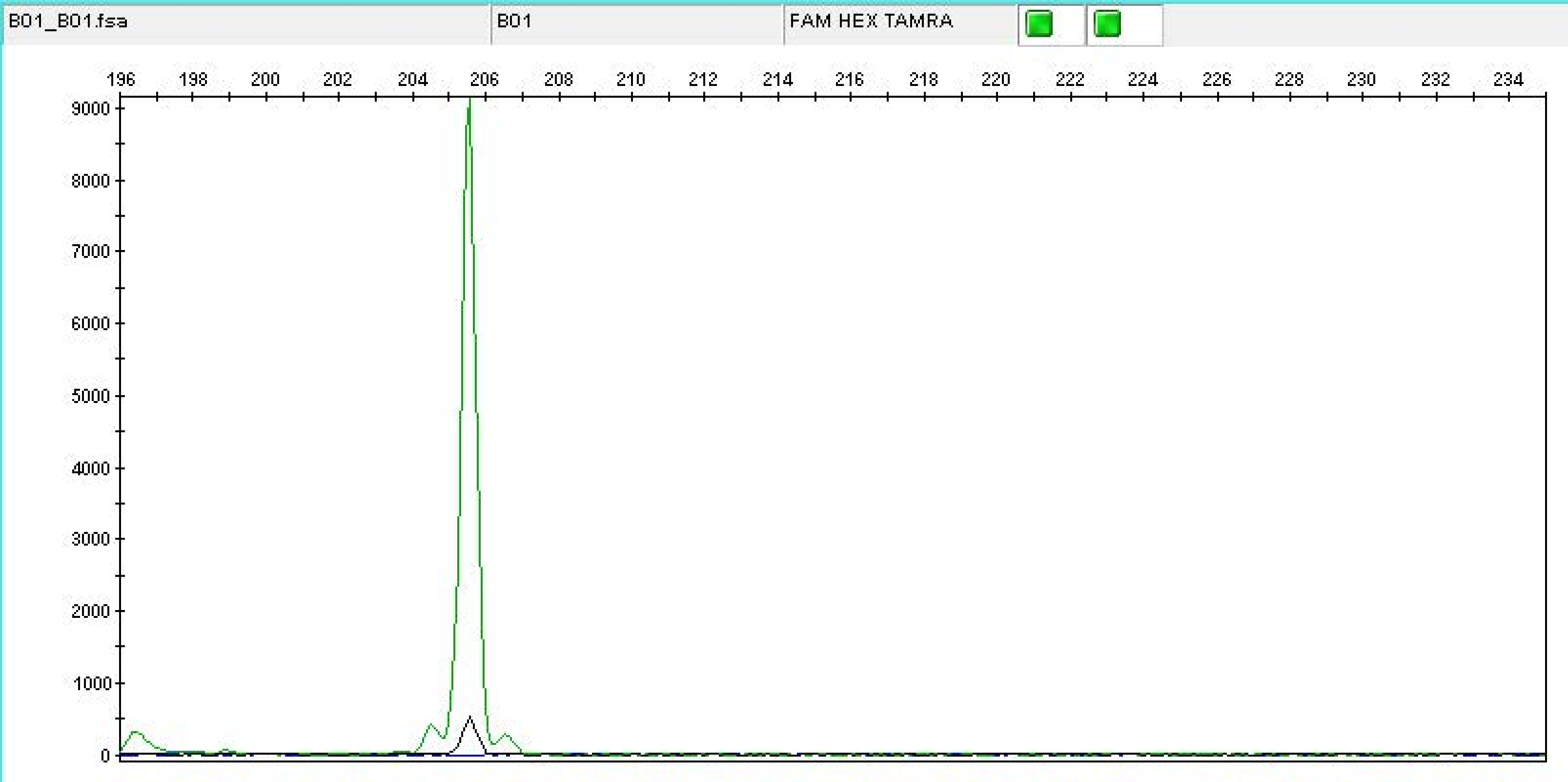

B01_B01.fsa
B01
FAM HEX TAMRA
196 198 200 202 204 206 208 210 212 214 216 218 220 222 224 226 228 230 232 234
9000
8000
7000
6000
5000
4000
3000
2000
1000
0

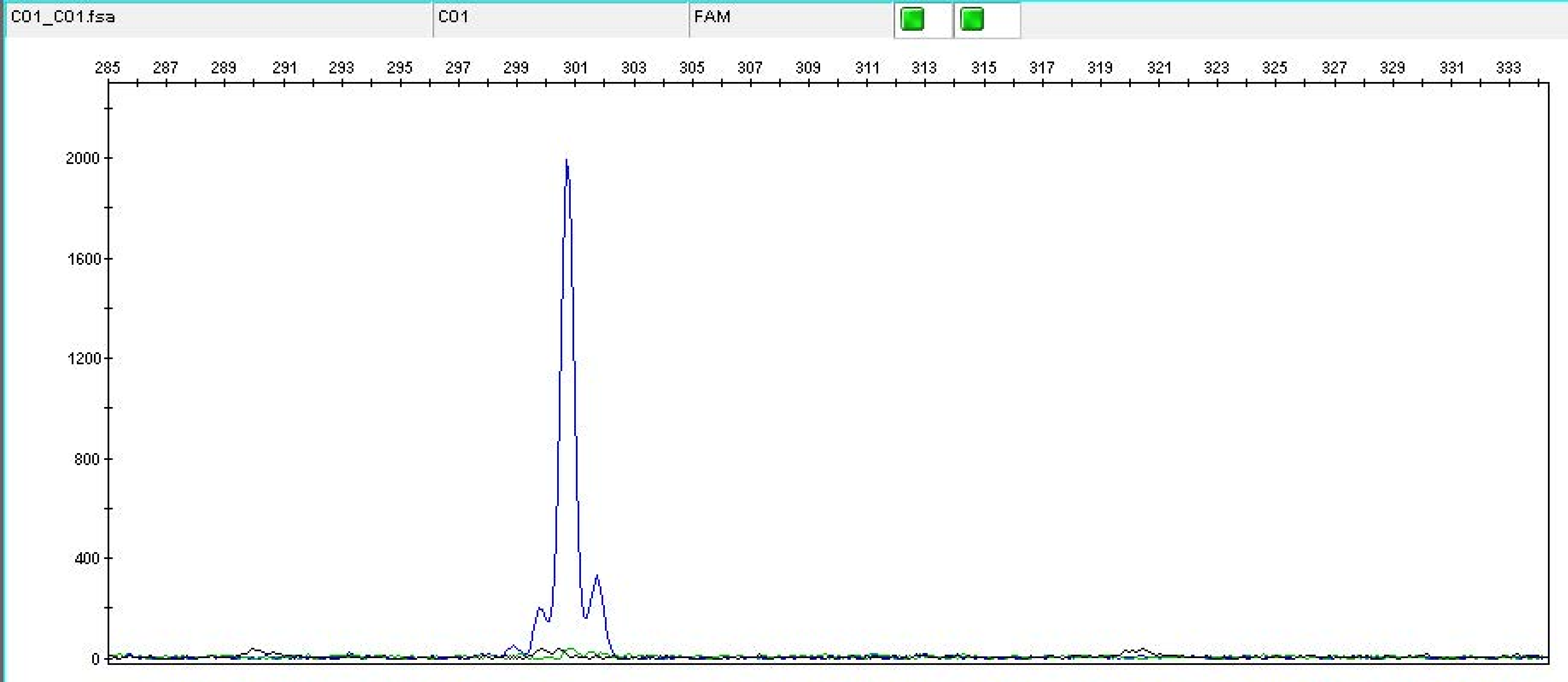
C01_C01.fsa
C01
FAM
285
287
289
291
293
295
297
299
301
303
305
307
309
311
313
315
317
319
321
323
325
327
329
331
333
2000
1600
1200
800
400
0

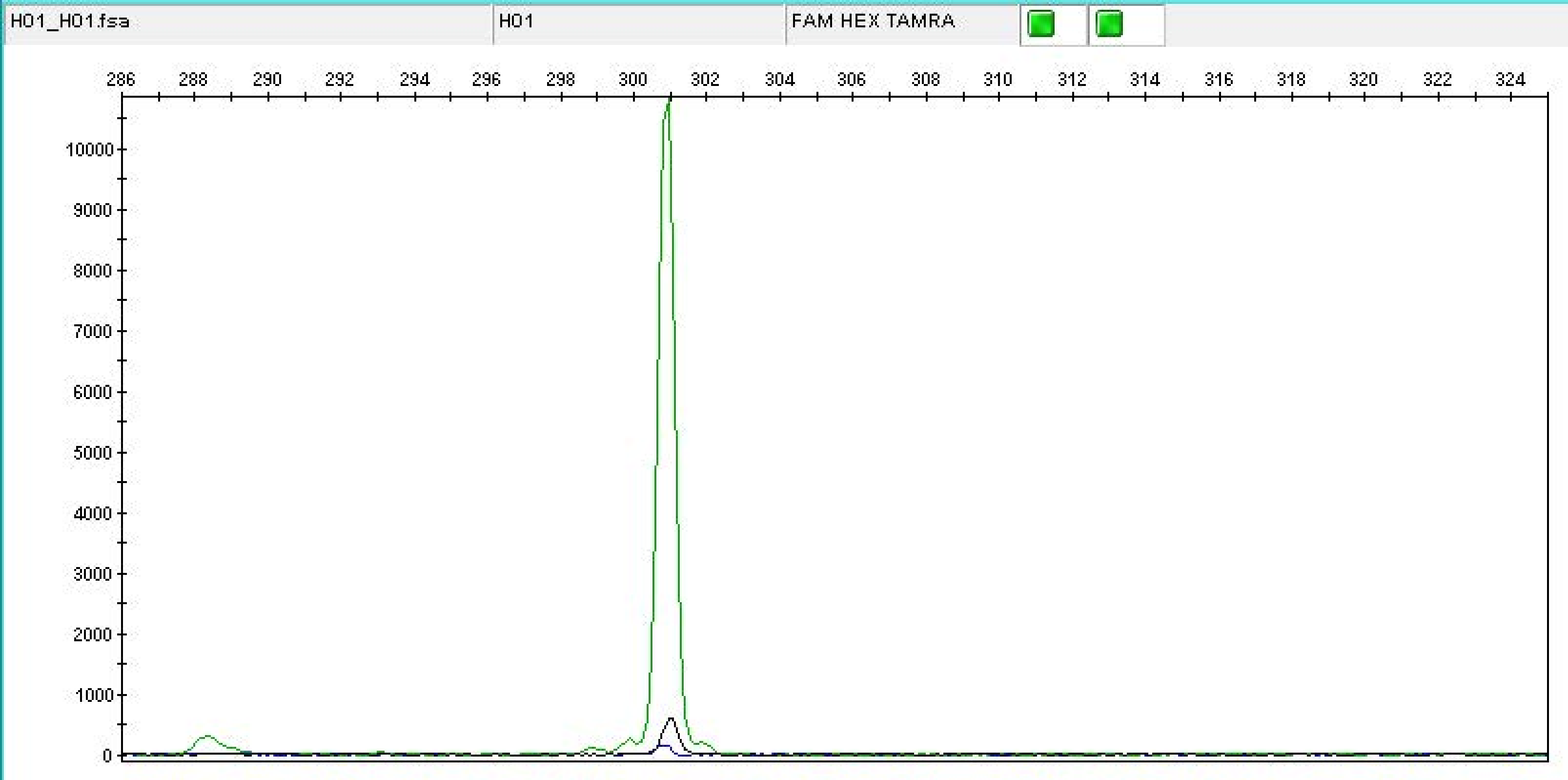
H01_H01.fsa
H01
FAM HEX TAMRA
286 288 290 292 294 296 298 300 302 304 306 308 310 312 314 316 318 320 322 324
10000
9000
8000
7000
6000
5000
4000
3000
2000
1000
0

G12_G12.fsa | G12 | FAM

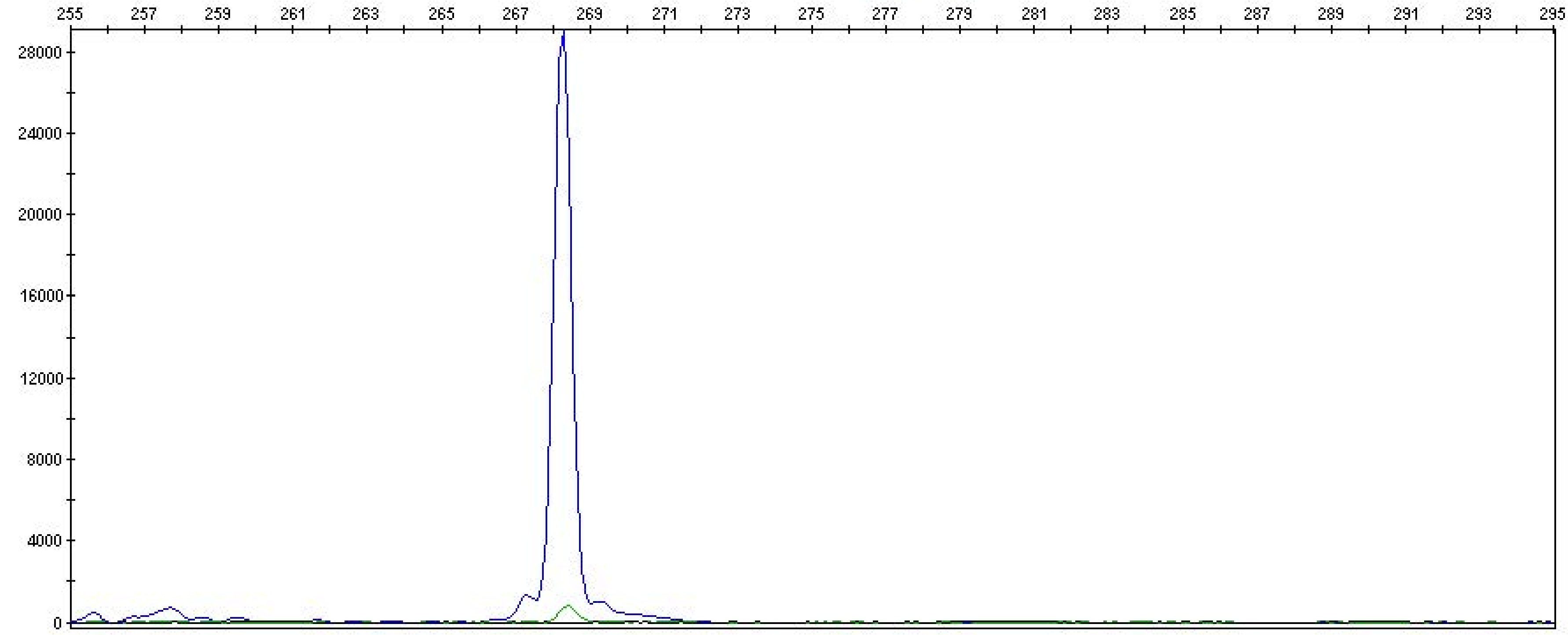

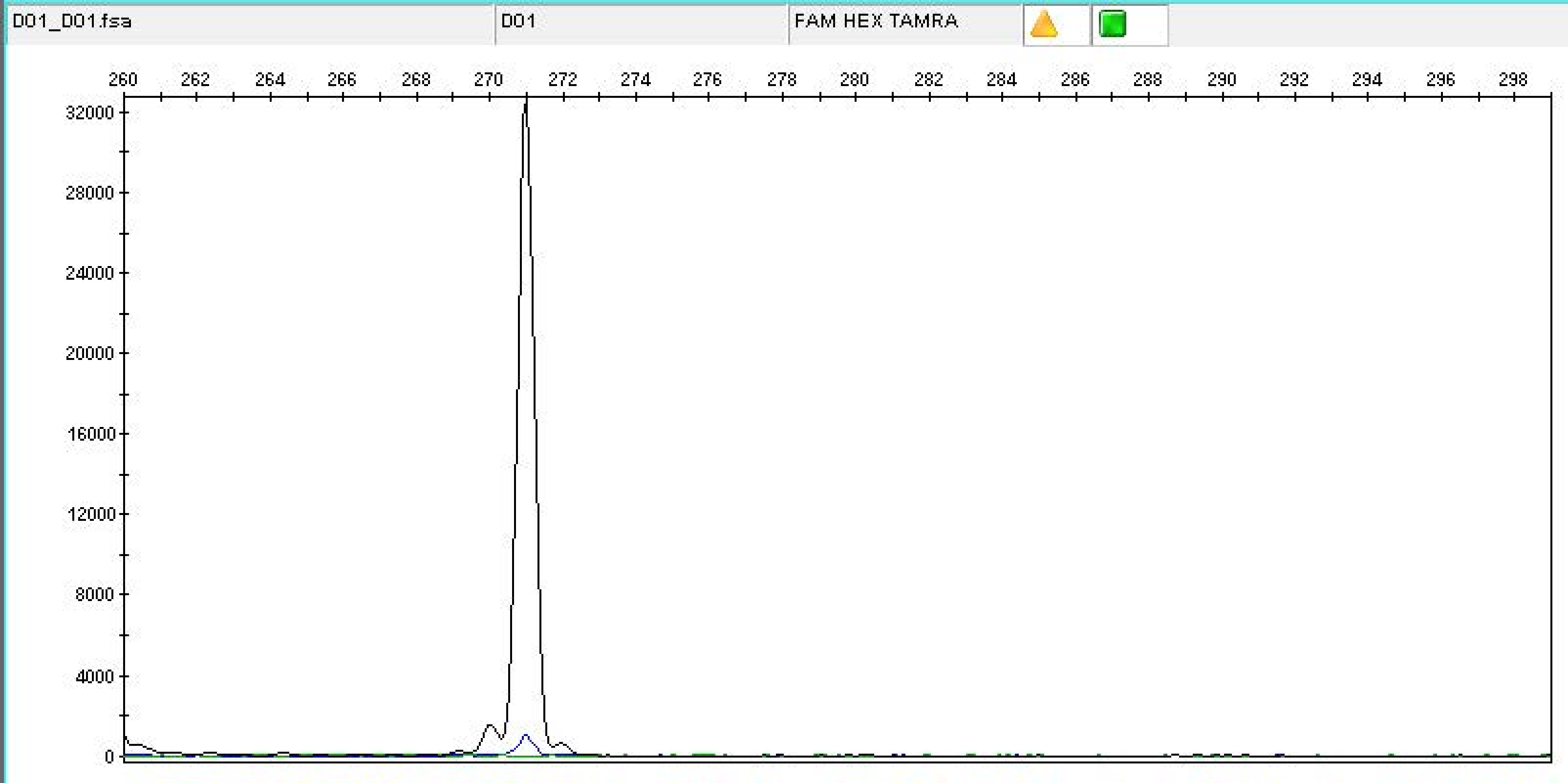

D01_D01.fsa
D01
FAM HEX TAMRA
260
262
264
266
268
270
272
274
276
278
280
282
284
286
288
290
292
294
296
298
32000
28000
24000
20000
16000
12000
8000
4000
0

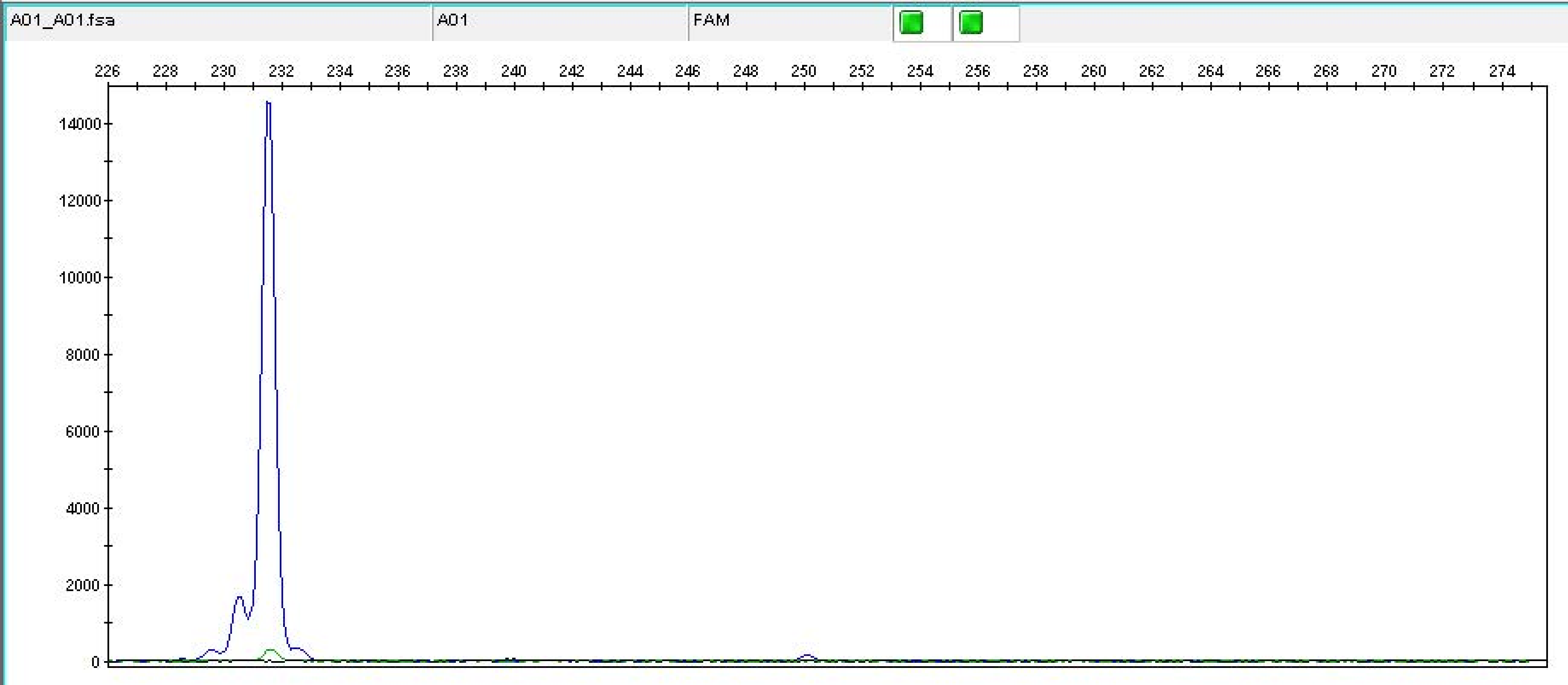

A01_A01.fsa
A01
FAM
226 228 230 232 234 236 238 240 242 244 246 248 250 252 254 256 258 260 262 264 266 268 270 272 274
14000
12000
10000
8000
6000
4000
2000
0

F01_F01.fsa	F01	FAM HEX TAMRA

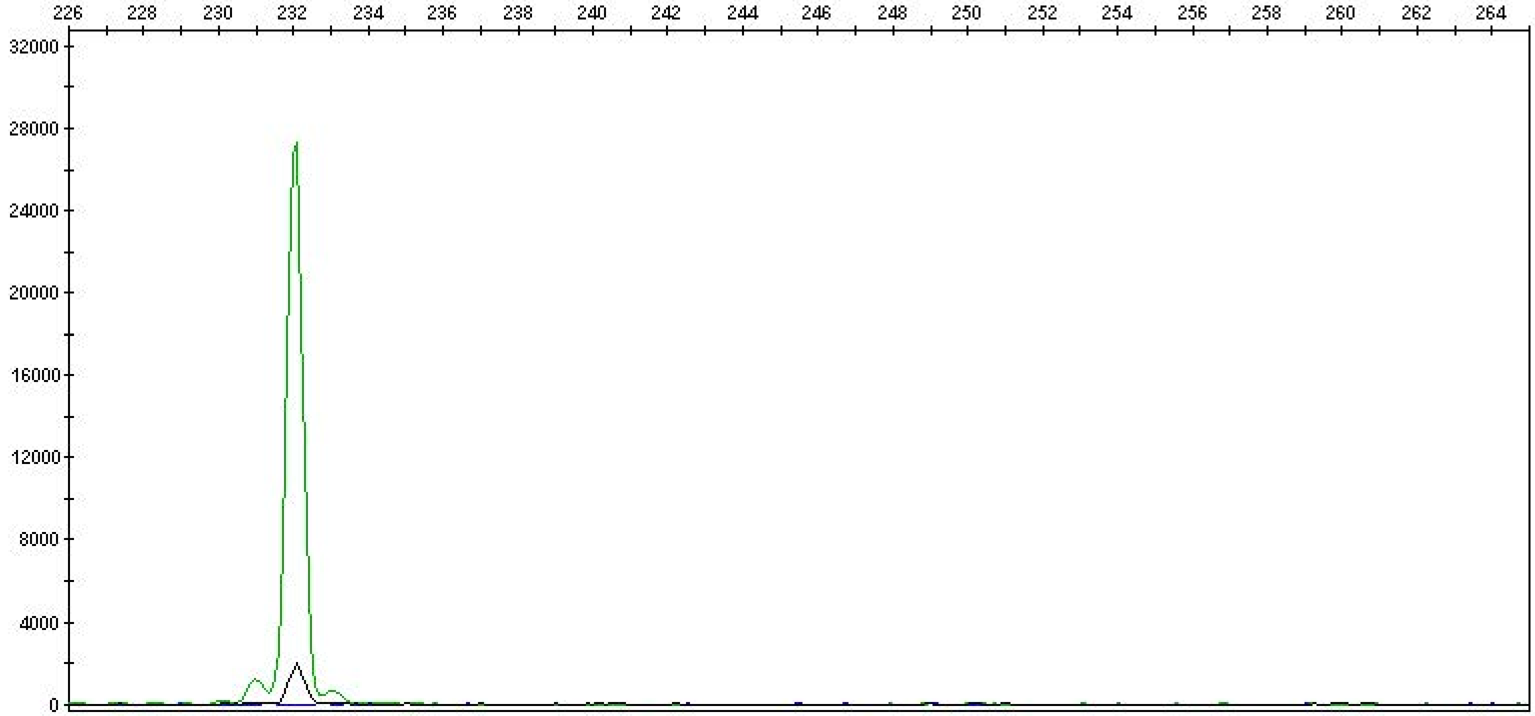

Supplement: Supplementary file 1 — Supplementary Information. [file 41598_2020_59986_MOESM1_ESM.pdf]
